# Intestinal epithelial NAIP/NLRC4 restricts systemic dissemination of the adapted pathogen *Salmonella* Typhimurium due to site-specific bacterial PAMP expression

**DOI:** 10.1038/s41385-019-0247-0

**Published:** 2020-01-17

**Authors:** Annika Hausmann, Desirée Böck, Petra Geiser, Dorothée L. Berthold, Stefan A. Fattinger, Markus Furter, Judith A. Bouman, Manja Barthel-Scherrer, Crispin M. Lang, Erik Bakkeren, Isabel Kolinko, Médéric Diard, Dirk Bumann, Emma Slack, Roland R. Regoes, Martin Pilhofer, Mikael E. Sellin, Wolf-Dietrich Hardt

**Affiliations:** 1 Institute of Microbiology, Department of Biology, ETH Zurich, Zurich, Switzerland; 2 Institute of Molecular Biology and Biophysics, Department of Biology, ETH Zurich, Zurich, Switzerland; 3 Science for Life Laboratory, Department of Medical Biochemistry and Microbiology, Uppsala University, Uppsala, Sweden; 4 Institute for Integrative Biology, Department of Environmental Science, ETH Zurich, Zurich, Switzerland; 5 Present Address: Science for Life Laboratory, Department of Medical Biochemistry and Microbiology, Uppsala University, Uppsala, Sweden; 6 Present Address: The Kennedy Institute of Rheumatology, University of Oxford, Oxford, UK; 7 Present Address: Biozentrum Basel, University of Basel, Basel, Switzerland; 8 Present Address: Institute for Food, Nutrition and Health, D-HEST, ETH Zürich, Zürich, Switzerland

## Abstract

Inflammasomes can prevent systemic dissemination of enteropathogenic bacteria. As adapted pathogens including *Salmonella* Typhimurium (*S*. Tm) have evolved evasion strategies, it has remained unclear when and where inflammasomes restrict their dissemination. Bacterial population dynamics establish that the NAIP/NLRC4 inflammasome specifically restricts *S*. Tm migration from the gut to draining lymph nodes. This is solely attributable to NAIP/NLRC4 within intestinal epithelial cells (IECs), while *S*. Tm evades restriction by phagocyte NAIP/NLRC4. NLRP3 and Caspase-11 also fail to restrict *S*. Tm mucosa traversal, migration to lymph nodes, and systemic pathogen growth. The ability of IECs (not phagocytes) to mount a NAIP/NLRC4 defense in vivo is explained by particularly high NAIP/NLRC4 expression in IECs and the necessity for epithelium-invading *S*. Tm to express the NAIP1-6 ligands—flagella and type-III-secretion-system-1. Imaging reveals both ligands to be promptly downregulated following IEC-traversal. These results highlight the importance of intestinal epithelial NAIP/NLRC4 in blocking bacterial dissemination in vivo, and explain why this constitutes a uniquely evasion-proof defense against the adapted enteropathogen *S*. Tm.

## Abstract (web summary)

▓

## Graphical abstract

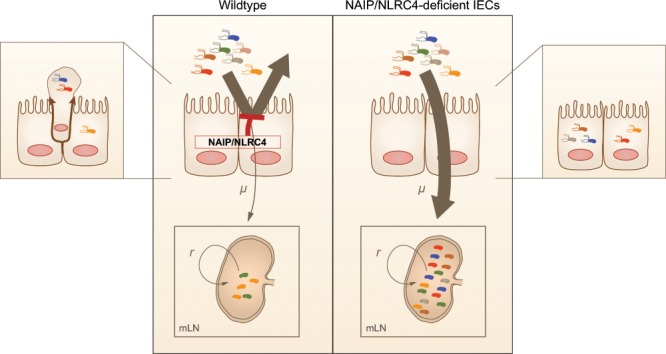

## Introduction

Oral bacterial infection causes localized gastrointestinal disease, but pathogen dissemination (from here on termed migration) to systemic sites can lead to life-threatening complications. Multiple host defenses therefore cooperate to limit mucosal infection and pathogen spread.^1^ The intestinal mucus lining and antimicrobial peptide secretion restrict mucosal invasion.^2–7^ Intestinal epithelial cells (IECs) and lamina propria phagocytes mount cell-intrinsic programs and release pro-inflammatory signals to counter pathogens that breach this first barrier.^2,8–11^ Diverse immune cell types also patrol systemic organs and prevent excessive pathogen replication.^12–15^ This multilayered host defense is triggered by pattern recognition receptors (PRRs) that detect pathogen-associated molecular patterns (PAMPs), exposed by invading microbes. However, it is not fully understood which innate defense mechanisms act at which stage of the infection in vivo and what quantitative impact can be assigned to each layer of the defense.

The prototypic enteropathogen *Salmonella enterica* Typhimurium (*S*. Tm) colonizes the gut lumen, invades the mucosa, and can migrate to mesenteric lymph nodes (mLN), spleen, and liver, causing life-threatening infection, e.g. in immunocompromised hosts.^1^ Luminal *S*. Tm expresses flagella to target gaps in the mucus layer,^4,16^ a type-III-secretion system (TTSS-1) to actively invade IECs,^17^ and, upon cell invasion, a second TTSS (TTSS-2) to promote IEC traversal.^18^ Mononuclear phagocytes (e.g., dendritic cells (DCs), macrophages) are involved in multiple steps of the *S*. Tm infection cycle. They facilitate *S*. Tm uptake across the epithelial barrier,^19–21^ lodge *S*. Tm within the lamina propria and at systemic sites,^18,22^ and act as vessels for *S*. Tm migration between organs.^23^ Consequently, restriction of systemic *S*. Tm infection may depend on the capacity of both IECs and phagocytes to recognize the pathogen through PRR(s) and mount appropriate counter measures. For an adapted pathogen like *S*. Tm, this task is complicated by the bacterium’s evolved ability to evade PRR recognition, through e.g., context-dependent regulation of its gene expression or inhibition of host-cell signaling.^1,24,25^

Inflammasomes are multimeric signaling complexes that assemble in the host cell cytosol upon sensing of PAMPs or cellular damage by PRRs of the Nod-like receptor family (NLRs; NLRP1, NLRP3, NLRC4), Aim2, or Pyrin.^26^ Inflammasome assembly causes cleavage of pro-inflammatory Caspase-1,^27^ secretion of interleukin-1 family cytokines and lipid mediators,^10,28^ and prompt cell death.^29^ During bacterial infection, these effects promote clearance of intracellular pathogens, elicit local inflammation, and foster recruitment of effector cells, e.g., neutrophils, to sites of infection.^14,30,31^ Work in cultured macrophages or DCs established several inflammasomes capable of responding to *S*. Tm infection. NAIP receptors (NAIP1, 2, 5, 6 in mice) recognize the TTSS-1 rod and needle proteins (NAIP1, 2) or flagellin (NAIP 5, 6) in the cytoplasm,^32–36^ and drive assembly of a NAIP/NLRC4 inflammasome. NLRP3 can also sense *S*. Tm infection,^37–39^ although the specific ligand(s) detected remains unknown. In addition, a non-canonical Caspase-4/11 inflammasome directly senses lipopolysaccharide (LPS) upon cytosolic escape of *S*. Tm from the endosomal compartment.^40–42^ Inflammasome activation upon *S*. Tm infection is not confined to phagocytes, but has also been shown to occur in epithelial cells, in IECs particularly involving NAIP/NLRC4^10,11^ or Caspase-4/11.^43^

The capacity of inflammasomes to restrict *S*. Tm migration and growth at systemic sites in vivo has been subject to much debate. Caspase-1/11-deficient mice showed either reduced or enhanced susceptibility to systemic *S*. Tm infection.^44–46^ NLRC4-deficiency enhanced susceptibility to systemic infection,^44,47,48^ but not in all mouse backgrounds.^8^ Moreover, *Nlrp3*^−/−^ mice exhibited identical disease kinetics as controls,^37,44,49^ but a functional redundancy between NLRP3 and NLRC4 during oral *S*. Tm infection has been proposed.^37,39^ Finally, Caspase-11 deletion had no impact on systemic *S*. Tm loads,^43,50^ but one study found higher *S*. Tm loads in the gut mucosa during late stage infection.^43^ It has become evident that separately held control animals develop a unique gut microbiota that can deviate considerably from the experimental group.^51–54^ This confounding factor may explain some of the discrepancies between early in vivo *S*. Tm infection studies.

In a littermate-controlled study of early *S*. Tm gut infection we identified IEC NAIP/NLRC4 as a key mucosal defense, which drives expulsion of infected IECs to limit mucosal tissue *S*. Tm loads.^11^ Protection by epithelial NLRC4 was confirmed in a subsequent study,^10^ and also pertains to *Citrobacter rodentium* infection.^55^ Importantly, mice globally lacking NAIP proteins also featured elevated systemic *S*. Tm loads upon oral challenge.^11^ Due to the central involvement of phagocytes in pathogen migration and growth at systemic sites, this raised the question if NAIP/NLRC4 within IECs, phagocytes, or both, restrict disseminated *S*. Tm infection. Moreover, it remained unclear if pathogen restriction also involved NLRP3 and/or Caspase-11 and whether redundancies between the inflammasomes exist in vivo.

Here, we applied a bacterial population dynamics approach and littermate-controlled infections of inflammasome-deficient mice to address which inflammasome(s) in which cell types restrict disseminated oral *S*. Tm infection. We find that *S*. Tm successfully escapes restriction by phagocyte inflammasomes that in principle can recognize the bacterium. By contrast, the necessity for *S*. Tm to express the NAIP ligands—flagella and TTSS-1—during the epithelial cell invasion step explains why intestinal epithelial NAIP/NLRC4 constitutes a unique restriction system that even this highly adapted pathogen cannot fully evade.

## Results

### NAIP/NLRC4 potently restricts *S*. Tm migration from the gut lumen to systemic sites

Our in vivo analysis of the NAIP/NLRC4-mediated defense focused on the *S*. Tm infection dynamics during the first 24h after orogastric inoculation in the Streptomycin pretreated mouse model.^56^ Due to its highly reproducible, fast and robust kinetics, this infection setup is ideally suited to study innate immune restriction of enteropathogen dissemination to systemic sites. Within 24 h, the pathogen colonizes the gut lumen of Streptomycin pretreated mice, invades the gut mucosa, and disseminates systemically via gut-draining mLN.^57^ The size of a pathogen population inside the mLN is the product of several parameters, i.e., bacterial immigration to this site, replication on the way to and within the organ, emigration to other sites and elimination of the pathogen by the host. In contrast to classical selective plating of infected organs, which merely provides a snapshot of the bacterial population size, infections with mixtures of wild-type isogenic tagged strains (WITS), combined with mathematical modeling can reveal the dynamic parameters and thereby provide essential information on pathogen restrictive mechanisms (Fig S1).^58–63^

**Fig. 1:**
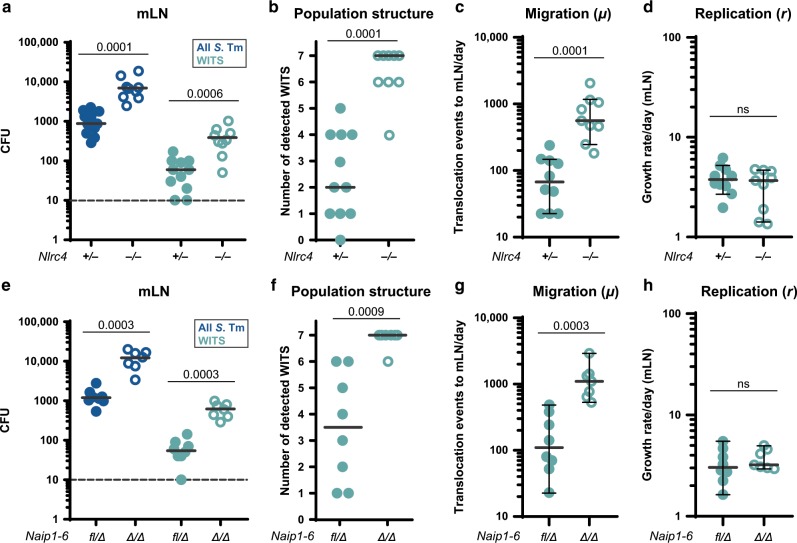
The NAIP/NLRC4 inflammasome restricts pathogen migration to the mLN during oral *S*. Tm infection. Streptomycin pretreated mice were orally infected with 5 × 10^7^ CFU *S*. Tm. **a** *S*. Tm counts (colony forming units, CFU) in the mLN at 24 hpi in *Nlrc4*^−/−^ mice (open circles) are ~10-fold increased compared to *Nlrc4*^+/−^ (circles) littermates. **b** The populations recovered from the mLN of *Nlrc4*^−/−^ mice (open circles) display higher variety in WITS than those isolated from *Nlrc4*^+/−^ (circles) littermates. Analysis of mice depicted in **a**. Only mice with detectable WITS (plating) in the mLN were included in the analysis, remaining samples were set to 0. **c** The migration rate *µ* from the cecal lumen (of mice shown in **b** with ≥1 WITS-CFU per mLN, translocation events/day) to the mLN of *Nlrc4*^−/−^ mice (open circles) is 8.7-fold increased compared to *Nlrc4*^+/−^ (circles) littermates. **d** *S*. Tm growth rate *r* within the mLN (of mice shown in **b** with ≥1 WITS-CFU per mLN, depicted as growth rate per day) is independent of NLRC4. **e** *S*. Tm counts in the mLN of *Naip1-6*^*Δ/Δ*^ mice (open circles) are ~10-fold increased at 24 hpi compared to *Naip1-6*^*fl/Δ*^ (circles) littermates. **f** The populations recovered from the mLN of *Naip1-6*^*Δ/Δ*^ mice (open circles) display higher variety in WITS than that of *Naip1-6*^*fl/Δ*^ (circles) littermates. Analysis of mice depicted in **e**. Only mice with detectable WITS (plating) in the mLN were included in the analysis, remaining samples were set to 0. **g** The migration rate *µ* from the cecal lumen (of mice shown in **f** with ≥1 WITS-CFU per mLN, translocation events/day) to the mLN of *Naip1-6*^*Δ/Δ*^ mice (open circles) is 6.7-fold increased compared to *Naip1-6*^*fl/Δ*^ (circles) littermates. **h** *S*. Tm growth rate *r* within the mLN (of mice shown in **f** with ≥1 WITS-CFU per mLN, depicted as growth rate per day) is independent of NAIP1-6. Depicted are counts of all *S*. Tm (dark blue, selected for with Streptomycin) and specifically of the WITS (light blue, selected for with Kanamycin, 5% of inoculum). Each circle represents one mouse. Combined data of three (**e**–**h**) or four (**a**–**d**) independent experiments. Dotted line: detection limit. Gray line: Median, in **c**, **d**, **g**, and **h** 95%-Confidence Intervals are indicated. Statistical analysis: Mann-Whitney-*U* Test, *p*-values indicated, ns: *p* ≥ 0.05.

To establish the quantitative infection parameters, we infected Streptomycin pretreated mice with a mixed inoculum (5 × 10^7^ total CFU per gavage), comprised of non-tagged *S*. Tm and seven WITS each spiked in at a 1:140 dilution (i.e., the WITS strains together made up 5% of the inoculum). By conventional plating, we detected ~10-fold increased total *S*. Tm loads in the mLN of *Nlrc4*^−/−^^64^ mice compared to their heterozygous littermate controls at 24 h post-infection (hpi) (Fig. 1a, “all *S*. Tm”). This phenotype was confirmed by selective plating of the WITS population (Fig. 1a, “WITS”). In line with earlier work establishing that gut luminal *S*. Tm loads are independent of gut inflammation during the first 24h,^65^ we did not detect any changes in luminal colonization (Fig S2A). To determine the cause of the elevated mLN pathogen loads, we analyzed the number of distinct WITS recovered from this organ. In the mLN of infected *Nlrc4*^−/−^ mice all, or close to all, of the seven WITS were recovered at 24 hpi. By striking contrast, *Nlrc4*^+/−^ littermate control mLN harbored on average only two WITS (Fig. 1b).

The higher diversity of WITS in the mLN of *Nlrc4*^−/−^ mice suggested that more bacteria seed the organ to give rise to the mLN-lodged pathogen population. To test this hypothesis, we applied a mathematical model for analysis of population dynamics, considering pathogen migration (*µ*) and net growth (*r*^59^; see materials and methods for details). This established that the *S*. Tm migration rate (*µ*) from the cecal lumen to the mLN was elevated by 8.7-fold in *Nlrc4*^−/−^ mice compared to controls (Fig. 1c). Notably however, the presence or absence of NLRC4 did not affect the pathogen's net replication rate (*r*) within the mLN (Fig. 1d). We repeated these experiments in *Naip1-6*^*Δ/Δ*^^66^ mice, which lack the receptors activating the NLRC4 inflammasome.^66^ These mice phenocopied *Nlrc4*^−/−^ animals, showing a 6.7-fold increase in *S*. Tm migration to the mLN, but a similar within-mLN pathogen replication rate as control animals (Fig. 1e–h, S2B).

Taken together, these findings show that the NAIP/NLRC4 inflammasome is of key importance for restricting *S*. Tm migration from the gut lumen to the mLN, but does not affect the pathogen's replication within this target organ. By inference, this indicates that phagocyte NAIP/NLRC4 is dispensable for controlling pathogen growth in the mLN. It remained to be established if IEC or phagocyte NAIP/NLRC4 could explain restriction of pathogen migration from the gut, and if additional inflammasomes also impact this process.

### NLRP3 and Caspase-11 are dispensable for control of *S*. Tm dissemination from the gut, even in the absence of NLRC4

**Fig. 2:**
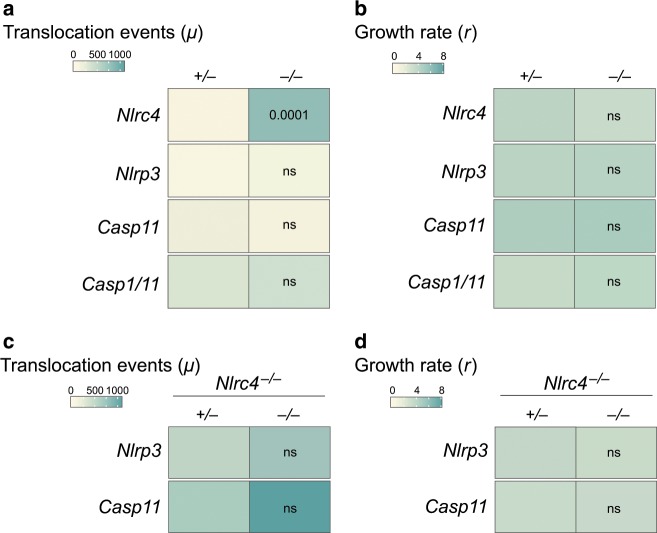
NLRP3 and Caspase-11 inflammasomes do not contribute to restriction of early *S*. Tm migration to the mLN. **a** *S*. Tm migration rate *µ* (of mice shown in Fig S3A-C with ≥1 WITS-CFU per mLN, translocation events per day) is independent of NLRP3, Caspase-11, and Caspase-1. Data for *Nlrc4*^+/−^ and *Nlrc4*^−/−^ mice replotted from Fig. 1c for reference. **b** *S*. Tm growth rate per day *r* within the mLN (mice shown in Fig S3A-C with ≥ 1 WITS-CFU per mLN) is independent of NLRP3, Caspase-11, and Caspase-1. Data for *Nlrc4*^+/−^ and *Nlrc4*^−/−^ replotted from Fig. 1d for reference. Even in the absence of NLRC4, *S*. Tm migration rate *µ* (mice shown in Fig S3D-E with ≥1 WITS-CFU per mLN, translocation events per day, (**c**) and *S*. Tm growth rate per day *r* within the mLN (mice shown in Fig S3D-E with ≥1 WITS-CFU per mLN, (**d**) are independent of NLRP3 and Caspase-11. Combined data of three (*Nlrp3*,^−*/*−^ *Casp11*,^−*/*−^ *Casp11xNlrc4*^−*/*−^) or four (*Nlrc4*,^−*/*−^ *Casp1/11*,^−*/*−^ *Nalp3xNlrc4*^−*/*−^) independent experiments. Statistical analysis: Mann–Whitney-*U* Test, p-values indicated, ns: *p* ≥ 0.05.

The significance of the NLRP3 inflammasome and the non-canonical Caspase-11 inflammasome during oral *S*. Tm infection and their role in limiting pathogen levels at systemic sites are ambiguous.^11,37,39,43,49,50^ To quantitatively assess the involvement of these inflammasomes, we infected *Nlrp3*^−/−^^67^ and *Casp11*^−/−^^41^ mice using the experimental setup described in Fig. 1. We did not observe any difference between the total bacterial loads in the cecal lumen and mLN, the WITS loads, or the numbers of WITS recovered from the mLN of either knockout line, when compared to their respective littermate controls (Fig S2C-D, S3A-B). Moreover, mathematical inference confirmed equivalent values for migration (*µ*) and net replication (*r*) in knockouts and controls (Fig. 2a, b; values for *Nlrc4*^−/−^ mice replotted from Fig. 1c, d for comparison). Hence, neither NLRP3, nor Caspase-11, impact the dynamic parameters of oral *S*. Tm dissemination. *Casp1/11*^−/−^^68^ mice featured an intermediate phenotype with regards to total mLN pathogen loads, which were increased ~3-fold (Fig S3C), while luminal colonization (Fig S2E), migration and replication of the bacterium were not altered (Fig. 2a, b). These data agree with a previously reported partial, but non-absolute, dependence of NAIP/NLRC4 on Caspase-1.^10,11,69,70^

As NAIP/NLRC4 potently limits pathogen migration (Fig. 1), we reasoned that the lack of involvement of NLRP3 and Caspase-11 might be attributable to redundancies with this inflammasome. We thus crossed *Nlrc4*^−/−^ mice with *Nlrp3-* or *Casp11-*knockout animals to obtain *Nlrc4*^−/−^*Nlrp3*^+/−^ and *Nlrc4*^−/−^*Nlrp3*^−/−^ littermates, as well as *Nlrc4*^−/−^*Casp11*^+/−^ and *Nlrc4*^−/−^*Casp11*^−/−^ littermates, for infections. However, also in an *Nlrc4*^−/−^ background, neither *Nlrp3-*ablation nor *Casp11*-ablation affected pathogen migration (*µ*) to the mLN, pathogen replication (*r*), total *S*. Tm loads in this organ by 24 hpi (Fig. 2c, d, Fig S3D-E), or cecal luminal growth (Fig S2F-G). In line with these data, we did not detect significant differences in mucosal inflammation between the mice (Fig S4). Thus, we conclude that in our oral infection model, NLRP3 and Caspase-11 are not involved in the control of *S*. Tm dissemination from the gut lumen during the first day of infection. As our approach monitors all infection steps from the gut lumen to the mLN, these data should exclude an impact of IEC and phagocyte NLRP3 and Caspase-11, alike.

### NAIP/NLRC4 in IECs, not in phagocytes, restricts systemic spread of *S*. Tm from the gut lumen

**Fig. 3:**
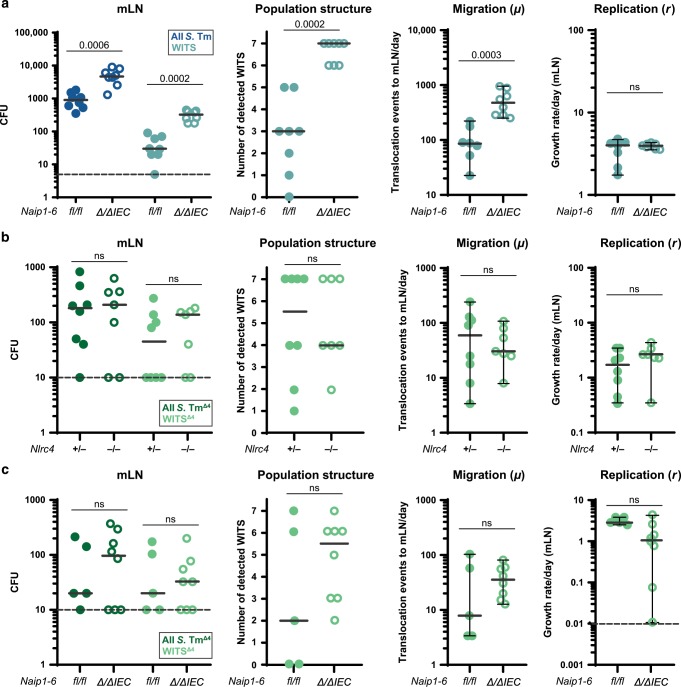
Intestinal epithelial NAIP/NLRC4 restricts pathogen migration to the mLN. **a** Streptomycin pretreated mice were orally infected with 5 × 10^7^ CFU *S*. Tm. *S*. Tm counts in the mLN at 24 hpi, number of WITS tags in the mLN and *S*. Tm migration rate *µ* to the mLN were significantly increased in *Naip1-6*^*Δ/ΔIEC*^ mice (open circles) compared to *Naip1-6*^*fl/fl*^ littermates (circles) and phenocopied *Naip1-6*^*Δ/Δ*^ mice (compare Fig. 1e–h). Growth rate *r* within the mLN was independent of NAIP1-6 within IECs. **b** Streptomycin pretreated mice were orally infected with 5 × 10^7^ CFU *S*. Tm^Δ4^. *S*. Tm^Δ4^, which bypasses IECs, is unaffected by NLRC4-mediated restriction during migration to and colonization of the mLN. This extends to epithelial NAIP1-6 (**c**). Depicted are counts of all *S*. Tm (dark blue, selected for with Streptomycin) and specifically of the WITS (light blue, selected for with Kanamycin, 5% of the inoculum) or *S*. Tm^Δ4^ (dark green, selected for with Streptomycin) and specifically of the WITS^Δ4^ (light green, selected for with Kanamycin, 33.3% of the inoculum). Each circle represents one mouse. Combined data of two (**b, c**) or three (**a**) independent experiments. Dotted line: detection limit. Gray line: Median, for *µ* and *r*, 95%-Confidence Intervals are indicated. Statistical analysis: Mann-Whitney-*U* Test, *p*-values indicated, ns: *p* ≥ 0.05.

The population dynamics analysis revealed that NAIP/NLRC4 restricts migration (*µ*) of *S*. Tm from the intestinal lumen to the mLN, but appears dispensable for containment of pathogen replication (*r*) within the mLN. The parameter *µ* in the model summarizes two main steps of the infection process: (i) the invasion/translocation of *S*. Tm across the cecal mucosa and (ii) the subsequent transport of the pathogen from the mucosa into the mLN. To specify which of the two components is impacted by NAIP/NLRC4, and in which cell type the restriction takes place, we infected mice lacking the NAIP receptors specifically in IECs (*Naip1-6*^*Δ/ΔIEC*^). Surprisingly, we found that IEC-specific ablation of NAIPs was sufficient to reproduce the pathogen migration phenotype observed in full body *Naip1-6* knockouts (Fig. 3a, compare with Fig. 1), while luminal colonization was unaffected (Fig S2H). The replication parameter (*r*) remained unaffected in *Naip1-6*^*Δ/ΔIEC*^ animals (Fig. 3a), further supporting a role for IEC NAIP/NLRC4 specifically in preventing pathogen migration from the gut lumen.

Notably, the restriction of systemic bacterial loads by epithelial NAIP/NLRC4 was equally relevant in an infection with a different *S*. Tm strain (*S*. Tm^14028^; Fig S5A, S2L). This suggests that the epithelial inflammasome is of general relevance for protection against *S*. Tm strains.

Active epithelial invasion, most prominently in the cecum,^4^ is the main pathway by which *S*. Tm traverses the intestinal epithelium to reach the mLN in the Streptomycin mouse model. By contrast, passive pathogen transport via lymphoid follicles and/or gut lumen-sampling DCs accounts for only ~10% of the total transport.^19,57^ Due to the pronounced effect of the IEC NAIP/NLRC4 inflammasome, it remained unclear if this “alternative” sampling route for traversal might also be restricted by the NAIP/NLRC4 inflammasome. The *S*. Tm mutant *S*. Tm^*Δ4*^ (SL1344 *sipAsopBsopEsopE2*) lacks the TTSS-1-delivered effector proteins necessary for active invasion into IECs, but retains the TTSS-1 structural components sensed by NAIP/NLRC4.^71^ While being severely attenuated for IEC invasion, non-invasive mutants like *S*. Tm^*Δ4*^ can still traverse the epithelial barrier by the passive sampling route.^19^ Hence, this strain only rarely passes through IECs on the way from the gut lumen to the mLN, but instead promptly enters the lamina propria phagocyte compartment. This feature allowed us to specifically analyze the impact of phagocyte NAIP/NLRC4 on the pathogen migration rate. Towards this aim, we applied the same procedure as described in Fig. 1. We infected *Nlrc4*^−/−^ mice for 24h with a mixture of non-tagged *S*. Tm^*Δ4*^ and spiked in the seven barcoded WITS^*Δ4*^ strains at a 1:21 dilution (i.e., the WITS^*Δ4*^ strains together made up 33.3% of the inoculum). In line with this strain only migrating through the passive sampling pathway, the absolute mLN loads of *S*. Tm^*Δ4*^ were ~10-fold lower than in an infection with wildtype *S*. Tm (compare Fig. 3b with Fig. 1a) and no detectable mucosal pathology was induced at 24 hpi (Fig S4C). This is in line with earlier work indicating that *sipAsopBsopEsopE2*-mediated epithelium invasion is the key trigger of acute mucosal inflammation.^72,73^ Importantly, when infecting with *S*. Tm^*Δ4,*^, we did not observe any effect of the ablation of NLRC4 on the mLN infection dynamics and cecal lumen colonization (Fig. 3b, S2I). This was also the case for *Naip1-6*^*Δ/ΔIEC*^ mice (Fig. 3c, S2K). It is interesting to note that we observed a slight, but non-significant trend towards higher mLN counts in *Naip1-6*^*Δ/ΔIEC*^ mice. We suspect that this is attributable to some residual epithelial invasion capacity of *S*. Tm^*Δ4*^ ^19,74^ and/or uptake by M-cells.

Finally, to formally exclude the involvement of DC NAIP/NLRC4 in *S*. Tm restriction, we infected mice lacking the NAIP receptors specifically in CD11c^+^ cells (*Naip1-6*^*Δ/ΔCD11c*^) with wild type *S*. Tm as described above. In line with our previous observations, the ablation of *Naip1-6* in CD11c^+^ cells did not affect luminal colonization (Fig S2M), mLN pathogen loads, migration of *S*. Tm to the mLN and replication of the bacterium at this site (Fig S5B). Altogether, these data demonstrate that i) NAIP/NLRC4 specifically in IECs acts as a firewall against pathogen dissemination from the gut lumen, whereas ii) this inflammasome has minimal impact in phagocyte populations that take up *S*. Tm in the mucosa, transport the pathogen to the mLN, or lodge the bacteria within this site.^18,19,23^

### NLRC4, NLRP3 and Caspase-11 inflammasomes are all dispensable during early systemic *S*. Tm infection

**Fig. 4:**
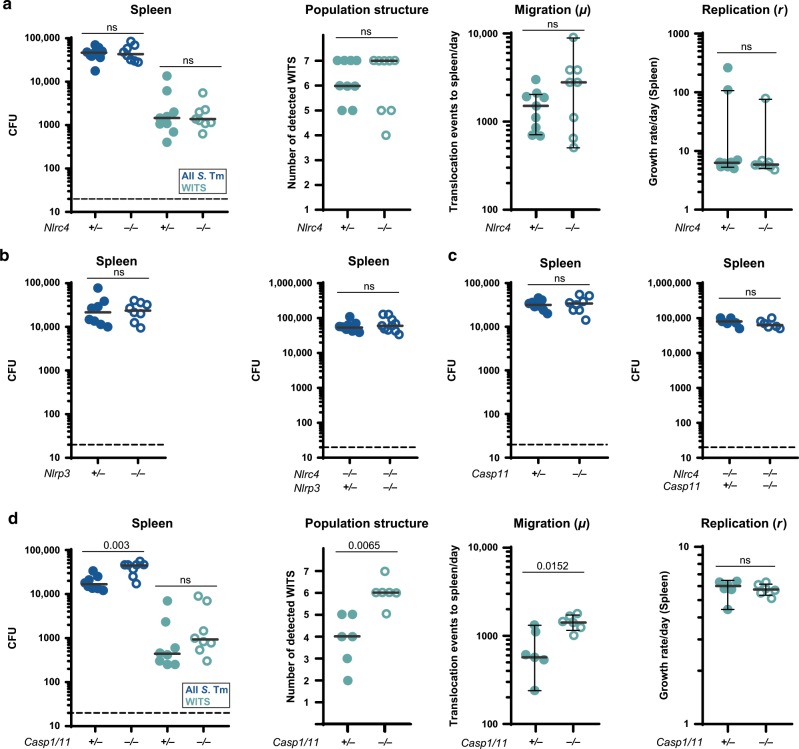
NLRC4, NLRP3 and Caspase-11 are dispensable for pathogen defense in a systemic infection model. Mice were infected iv with 10^4^ CFU *S*. Tm for 24 h. **a** *S*. Tm counts in the spleen at 24 hpi, number of WITS tags, *S*. Tm migration rate *µ* to and replication rate *r* within the spleen were not altered by ablation of NLRC4 (*Nlrc4*^+/−^ (circles) and *Nlrc4*^−/−^ (open circles)). **b** *S*. Tm counts in the spleen at 24 hpi were not altered in *Nlrp3*^−/−^ (open circles) compared to *Nlrp3*^+/−^ (circles) mice (left panel). This was also the case in an *Nlrc4*^−/−^ background (*Nlrc4*^−/−^*Nlrp3*^+/−^ (circles) and *Nlrc4*^−/−^ *Nlrp3*^−/−^ (open circles), right panel) mice. **c** *S*. Tm counts in the spleen at 24 hpi were not altered in *Casp11*^−/−^ (open circles) compared to *Casp11*^+/−^ (circles) mice (left panel), even in an *Nlrc4*^−/−^ background (*Nlrc4*^−/−^*Casp11*^+/−^ (circles) and *Nlrc4*^−/−^ *Casp11*^−/−^ (open circles), right panel). **d** *S*. Tm counts in the spleen, number of WITS tags and *S*. Tm migration rate *µ* in *Casp1/11*^−/−^ mice (open circles) are slightly increased at 24 hpi compared to *Casp1/11*^+/−^ littermates (circles), whereas the replication rate is not affected. Each circle represents one mouse. In **a** and **d** counts of all *S*. Tm (dark blue, selected for with Streptomycin) and specifically of the WITS (light blue, selected for with Kanamycin, 1% of the inoculum) are shown. Combined data of three (**a, b** *Nlrp3*,^−*/*−^ **c**) or four (**b** *Nlrp3xNlrc4*,^−*/*−^ *Casp1/11*^−*/*−^) independent experiments. Dotted line: detection limit. Gray line: Median, for *µ* and *r*, 95%-Confidence Intervals are indicated. Statistical analysis: Mann-Whitney-*U* Test, *p*-values indicated, ns: *p* ≥ 0.05.

As a more direct test for a possible involvement of phagocytes in inflammasome-mediated containment of *S*. Tm, we employed a systemic infection model. Here, *S*. Tm (10^4^ CFU) were applied intravenously (iv), resulting in a rapid uptake by phagocytes, and subsequent pathogen growth in the spleen and other systemic organs within 6-10 hpi.^58,75^ In this model, the main lymphoid organ infected during the first 2 days is the spleen, which was analyzed here (Fig. 4) at 24 hpi, analogously to the mLN in the oral infection model (Figs. 1–3). For estimation of *µ* and *r*, the inoculum was spiked with the seven barcoded WITS in a dilution of 1:700 (i.e., the WITS strains together made up 1% of the inoculum).

Strikingly, when analyzing spleen *S*. Tm loads in *Nlrc4*^−/−^ mice and littermate controls, we found NLRC4 to be completely dispensable for pathogen control. In line with this, we did not detect any differences in the number of WITS recovered from the spleen of these mice. Furthermore, the migration rate *µ* and the replication rate *r* within the spleen were not altered upon ablation of NLRC4 (Fig. 4a). We also observed no effect of NLRP3, or Caspase-11, on the containment of systemic *S*. Tm infection, even when analyzed in an *Nlrc4*^−/−^ background (Fig. 4b, c). *Casp1/11*^−/−^ mice again featured an intermediate phenotype, similar to our observations in the oral infection model, i.e.,  ~3-fold increased bacterial loads in the spleen by 24 hpi (Fig. 4d, compare to Fig S3C). Mathematical modeling suggests that this effect is due to a Caspase-1 mediated restriction of initial *S*. Tm migration to the spleen, rather than involvement in suppression of pathogen replication within this organ (Fig. 4d, population structure, *µ* and *r*). Hence, our data refute a significant impact of NLRC4, NLRP3 and Caspase-11 during early systemic *S*. Tm infection, while Caspase-1 contributes modestly to the control of pathogen loads. This resolves long-standing controversies in the literature about the involvement of different inflammasomes in host responses to wildtype *S*. Tm infection.^37,39,43,47,49,50,76^ It should be noted that differences in the studied infection time points or distinct virulence factor expression patterns of the employed *S*. Tm strains may also account for disparate phenotypes. Nevertheless, in our iv infections, Caspase-1 mediated defense appears independent of NLRC4, NLRP3, or Caspase-11. It remains to be established which other activation pathway might be involved.

### IECs express more *Naip* and *Nlrc4* transcripts than the remaining mucosal tissue cell types

To assess the NAIP/NLRC4 sensing potential of the specific cell types that interact with *S*. Tm during oral infection in mucosal and systemic tissues, we analyzed *Naip/Nlrc4* expression by quantitative PCR (qPCR). For assessment of *Naip* transcript levels in IECs vs. other cells of the mucosa (including phagocytes), and to set the baseline of the assay, we initially compared tissues from uninfected wildtype mice (*Naip1-6*^*fl/fl*^), to those of *Naip1-6*^*Δ/ΔIEC*^ and *Naip1-6*^*Δ/Δ*^ animals. Mice carrying just one intact allele of the *Naip1-6* locus (*Naip1-6*^*fl/Δ*^) served as an additional control for the sensitivity of the assay.

**Fig. 5:**
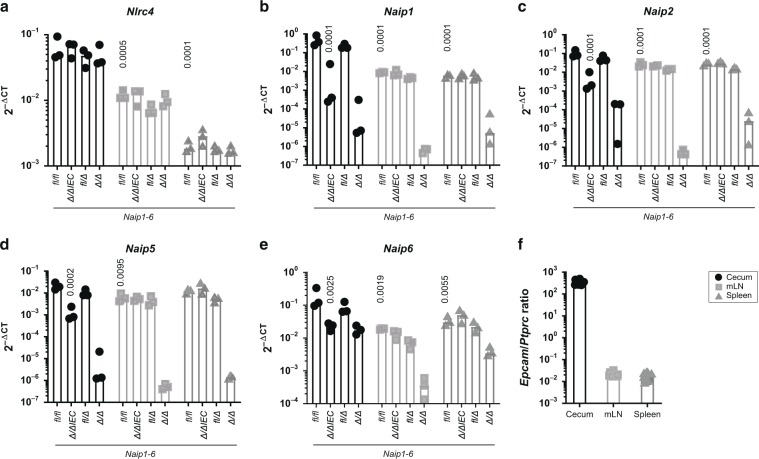
NAIP/NLRC4 inflammasome components are expressed in the cecal mucosa and in secondary lymphoid tissues. **a** *Nlrc4*, **b** *Naip1*, **c** *Naip2*, **d** *Naip5*, and **e** *Naip6* are expressed in the cecal mucosa, mLN and spleen. The cecal mucosa, and specifically IECs express high levels of *Naip* transcripts. Transcript levels of *Naip1-6*^fl/fl^, *Naip1-6*^*Δ/Δ*IEC^, *Naip1-6*^*Δ/fl*^, and *Naip1-6*^*Δ/Δ*^ mice depicted as 2^−ΔCT^ values. Expression levels were normalized to *Actb*. **f** For the samples depicted in **a**–**e**, expression levels of *Epcam* as an epithelial cell marker, and *Ptprc*, as a marker for immune cells, were compared to estimate the relative contribution of both cell types to the transcripts within the respective tissue. *n* = 3, each symbol represents one mouse. Median plotted. Statistical analysis: two-way ANOVA with Tukey`s correction. Selected *p*-values indicated in comparison to levels in cecal tissue of *Naip1-6*^fl/fl^ animals.

*Nlrc4* and *Naip1, 2, 5,* and *6* expression levels were markedly higher in the cecum tissue, as compared to both mLN and spleen (Fig. 5a–e, data from *Naip1-6*^*fl/fl*^ mice). *Nlrc4* was expressed at ~10-fold higher levels in the cecal mucosa than in the mLN and around 100-fold higher than in the spleen (Fig. 5a). Similar differences could be observed for the *Naip* transcripts. Especially *Naip1* was strongly expressed in the cecum, but ~100-fold reduced in the spleen (Fig. 5b). The bulk of the cecal *Naip1, 2, 5*, and *6* expression was attributable to IECs, in agreement with earlier work by us and others^11,77^ (Fig. 5b–e, compare *Naip1-6*^fl/fl^ with *Naip1-6*^*∆/∆IEC*^). Interestingly, when we analyzed the liver as a non-barrier, non-lymphoid organ, we found that the expression levels of *Nlrc4* and *Naip1-6* were generally ~100-fold lower than in the cecal mucosa (Fig S6A-B, compare to Fig. 5a–e). Hence, the cecal epithelium appears to be particularly loaded with NAIP/NLRC4 for detection of invading pathogens. This charging of non-immune cells with inflammasome receptors might be particularly pronounced in barrier tissues that are in close contact with microbes and engage actively in defense.^10,11,43,55,78^

In addition to the major epithelial *Naip* transcript pool, we noted a significant contribution from other cell types to the expression of the *Naip* genes in the cecal mucosa (Fig. 5b–f, compare *Naip1-6*^*Δ/ΔIEC*^ and *Naip1-6*^*Δ/Δ*^ animals). From the available dataset we could not infer to what percentage various immune cells, fibroblasts, endothelium and/or other cell types contribute to this non-IEC expression. To specifically analyze the expression of *Naip/Nlrc4* in intestinal DCs—the cell type that has been reported to take up *S*. Tm in the cecal mucosa and transport it to the mLN^19,23^—we sorted intestinal DC subsets from wildtype LPS-injected mice and PBS vehicle controls. Whereas *Nlrc4* was expressed equally in all intestinal DC subsets, CD103^+^ DCs expressed *Naip1* and *Naip2* at increased levels compared to other subsets (Fig S6C). The expression of these receptors was not boosted by exposure to the pro-inflammatory stimulus LPS. In fact, LPS-priming rather led to a downregulation of expression in some of the DC subsets, which might be explained by the immuno-tolerant phenotype of mucosal myeloid cells.^79^ In addition, this downregulation might represent a protective mechanism by which self-destruction of infected DCs is prevented to ensure antigen presentation.

Taken together, our data highlight that IECs express particularly high levels of *Naip* and *Nlrc4* transcripts, whereas DCs encountered by *S*. Tm subsequent to epithelial traversal and/or upon passive sampling appear to express more modest levels. Importantly though, the failure of DCs to restrict *S*. Tm dissemination through NAIP/NLRC4 in vivo cannot be explained by a complete lack of this inflammasome. However, the reduced expression levels (compared to IECs) might explain why *S*. Tm^*Δ4*^ dissemination is not efficiently restricted by NAIP/NLRC4 (Fig. 3).

### *S*. Tm expresses the NAIP ligand structures TTSS-1 and flagella during IEC invasion, but promptly downregulates them upon transit to the lamina propria and systemic tissues

*S*. Tm migration to systemic sites is potently restricted by IEC NAIP/NLRC4, but apparently not by phagocyte NAIP/NLRC4, even though both cell types express detectable levels of the required receptors (Figs. 3–5; Fig. S6C). One reason for this discrepancy might reside in the pathogen's gene expression program, which may prevent PAMP expression at certain sites. Such compartment-specific down regulation has been observed previously in various infection models.^25,80–82^ *S*. Tm requires TTSS-1 and flagella (composed of flagellin subunits) for the initial colonization and establishment of gut infection.^16,72^ Especially for invasion of IECs, which strongly express *Naip1*, *2*, *5*, *6*, and *Nlrc4* (Fig. 5a–e), the NAIP ligand structures TTSS-1 (for the delivery of the effector proteins SipA, SopB, SopE, and SopE2)^72,73^ and flagella (to subvert gaps in the mucus layer and reach the epithelium by directed motility)^4,16^ are both crucial. This could explain why infection events are efficiently sensed and restricted specifically by IEC NAIP/NLRC4. However, it remained unclear to which extent *S*. Tm regulates TTSS-1 and flagella expression during and after epithelium traversal.

**Fig. 6:**
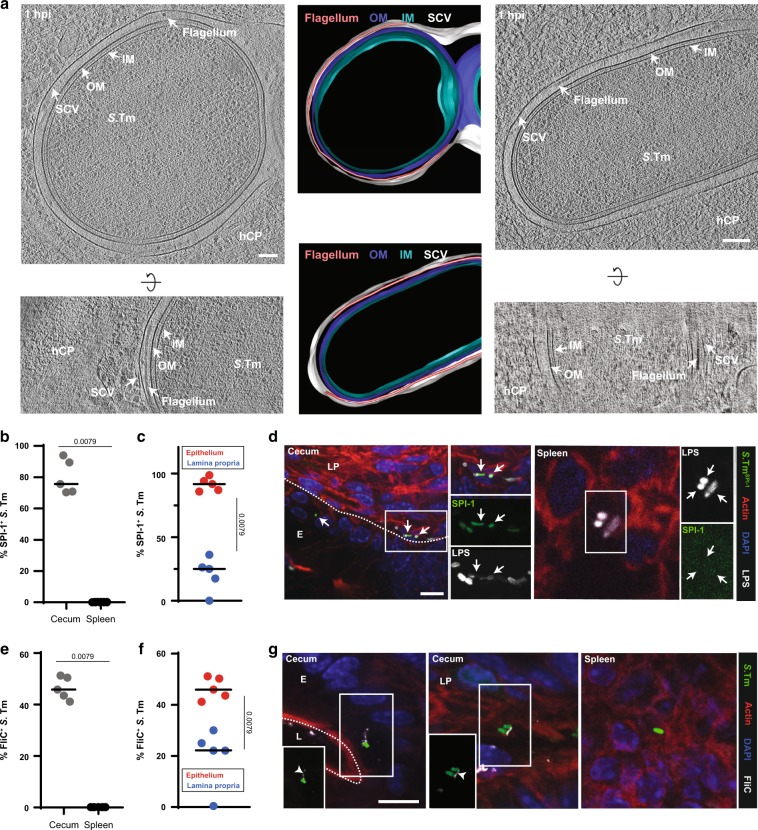
*S*. Tm expresses PAMPs recognized by NAIP/NLRC4 in the cecal epithelium, but downregulates them upon traversal. **a** *S*. Tm with potentially detached (left/upper panel) and attached (right/lower panel) flagella can be found inside SCVs. Shown are 15 nm slices of cryotomograms of cryo-FIB milled HeLa cells and the respective segmentations (representative for six tomograms; frozen at 1 hpi and MOI 300). OM: outer membrane. IM: inner membrane. SCV: *Salmonella* containing vacuole. hCP: host cell cytoplasm. Scale bar, 100 nm. **b** *S*. Tm as a rule expresses SPI-1 in the cecum (12 hpi, n^total *S*. Tm^=322) and downregulates the expression in the spleen (3 dpi, n^total *S*. Tm^=35) of *JH*^−/−^ mice. **c** The fraction of SPI-1^+^ *S*. Tm within the cecal mucosa decreases upon traversal to the lamina propria (n^total *S*. Tm epithelium^=253; n^total *S*. Tm lamina propria^=69). Quantifications shown in **b** and **c** are based on fluorescence microscopy of *S*. Tm^SPI-1-GFP^ expressing cytoplasmic GFP under the *prgH*-promotor and an LPS immunostaining. **d** Representative images of tissue sections (cecum, spleen), which were used for the quantifications in **b** and **c**. **e** Flagella are prominent in the cecal mucosa (12 hpi, n^total *S*. Tm^=341), while they are completely absent in the spleen (3 dpi, n^total *S*. Tm^=50) of *JH*^−/−^ mice. Quantifications were performed by fluorescence immunostaining of FliC. **f** Similar to SPI-1 (**c**) expression, flagella are less evident in the lamina propria (n^total *S*. Tm lamina propria^=39) than within IECs (n^total *S*. Tm epithelium^=302). **g** Representative images of tissue sections (cecum, spleen), which were used for the quantifications in **e** and **f**. White arrowheads: flagella; white arrows: SPI-1-GFP^+^ *S*. Tm (epithelium and lamina propria); E: epithelium; L: lumen; LP: lamina propria. Black bar: Median. Statistical analysis: Mann-Whitney-*U* Test, *p*-values indicated. Scale bars, 10 µm.

To clarify this aspect, we assessed the expression of NAIP-activating *S*. Tm PAMPs during the infection process. Initially, we optimized staining procedures in *S*. Tm infections of cultured HeLa epithelial cells, a process that relies on TTSS-1 (TTSS-1-deficient mutants ~500–1000-fold attenuated)^74^ and flagella-driven motility.^16^ While HeLa cells are clearly a simplistic test system riddled by genetic drift^83^ they nevertheless provide an efficient test system for establishing microscopy analysis pipelines. Notably, when infected HeLa cells were immunostained for the flagella subunit FliC, we could detect a significant fraction of FliC^+^ *S*. Tm also within the epithelial cells (Fig S7A). Notably, ~30% of the bacteria still stained positive for FliC at 7 hpi (Fig S7B). This is in line with previous work.^84^ These findings were supported by parallel analyses of infected HeLa cells using cryo-focused ion beam (cryo-FIB) milling and cryo-electron tomography (cryoET). Cryotomograms showed fully assembled flagella located between the bacterial surface and the membrane of the *Salmonella* containing vacuole (SCV) (Fig. 6a).

Previous work has shown that a subfraction of *S*. Tm may escape the SCV over time and become cytosolic.^81,85^ When we analyzed the cytosolic subpopulation with the help of *S*. Tm^localizer^, carrying a reporter for cytosolic escape (pCK100, glucose-6-phosphate (Glc6P)-driven expression of mCherry),^86–89^ we found that at 7 hpi, around one quarter of all the cytosolic bacteria was FliC^+^ (Fig S7B). Strikingly, we frequently detected bacteria with protruding flagella (Fig S7A, S7C). Cryotomograms, however, indicated a tight enclosure of both bacterium and flagellum within the SCV (Fig. 6a). Indeed, cytosolic localization correlated with a protruding flagellum conformation (Fig S7C). These data suggest that in addition to the mandatory expression of TTSS-1, a significant fraction of *S*. Tm carry flagella within cultured epithelial cells, and maintain or re-express these during egress into the cytosol, which is in line with observations by Knodler et al..^84^ These cytosolic *S*. Tm carrying the flagellum might be a potent trigger of epithelial inflammasome activation.

Results from immortalized cell lines exhibit limited reproducibility to in vivo settings due to altered cell physiology and lack of tissue environment.^83,90,91^ We therefore assessed if our observations also apply in vivo during IEC infection in the mouse model. We infected mice orally with *S*. Tm carrying a transcriptional reporter (*prgH-GFP*) for TTSS-1 expression (*S*. Tm^SPI-1-GFP^) and analyzed the pathogen populations in the cecum tissue (12 hpi) and at systemic sites (spleen, 3 dpi to obtain sufficient bacterial loads). Fluorescence microscopy was used to assess TTSS-1 expression (i.e., *prgH*-*GFP*) and flagella expression (staining with anti-FliC antibodies). Around 80% of the *S*. Tm lodged in IECs were SPI-1^+^ (Fig. 6b–d). This sharply contrasted to SPI-1 expression in the lamina propria (~20% SPI-1^+^ *S*. Tm) and the spleen (no detectable SPI-1 expression) (Fig. 6b–d). In line with the tissue culture data above, ~50% of the *S*. Tm lodged in the cecal epithelium also stained positive for FliC (Fig. 6e–g). This population was increased two-fold in the absence of epithelial NAIP receptors (Fig S8A), which correlated with a significantly increased fraction of cytosolic bacteria, especially with regard to microcolonies consisting ≥4 bacterial cells, in *Nlrc4*^−/−^ mice compared to *Nlrc4*^+/−^ or wildtype mice (Fig S8B). These observations are consistent with NLRC4/NAIP-dependent expulsion of IECs containing flagellated *S*. Tm. By contrast, we detected significantly less FliC^+^ *S*. Tm in the lamina propria (Fig. 6f, g) and no FliC^+^ bacteria in the spleen (Fig. 6e, g). It is currently not entirely clear if fully assembled flagella structures can activate the NAIP/NLRC4 inflammasome. Nevertheless, one can take the flagella as a proxy for the presence of flagellar subunits. Assuming that 90% of the lamina propria-lodged pathogens have reached this site by traversing the IECs (which necessitates the expression of PAMPs recognized by NAIP/NLRC4), our data indicate that the pathogen promptly down regulates expression of both structures during transit, thereby evading recognition by phagocyte NAIP/NLRC4.

To formally test if PAMP down regulation explains *S*. Tm evasion of phagocyte NAIP/NLRC4-restriction, we analyzed the effect of forced PAMP expression. This strategy has been used in the past to assess the PAMP-specificity of individual inflammasomes.^92^ To this end, we infected *Nlrc4*^+/−^ and *Nlrc4*^−/−^ littermate mice iv with a 1:1 mix of *S*. Tm^ECV^ (*S*. Tm^*ΔflgB TAG13*^ (pZ2500); empty control vector) and *S*. Tm^*fliC*ind^ (*S*. Tm^*ΔflgB TAG1*^ (pEM087); expression of FliC under a Doxycycline-inducible promoter^14^). We induced FliC expression by iv Doxycycline administration at 17 hpi, a time point when most of the bacteria should be lodged within splenic phagocytes. In NLRC4 proficient hosts, splenic *S*. Tm^*fliC*ind^ loads were ~10-fold lower than those of *S*. Tm^ECV^ by 24 hpi (Fig S9). Notably, this phenotype was not observed in *Nlrc4*^−/−^ mice (Fig S9). Thus, splenic phagocytes are able to sense *S*. Tm via the NAIP/NLRC4 inflammasome, but *S*. Tm as a rule avoids recognition. This further supports that downregulation of PAMP expression upon traversal of the gut epithelium contributes to *S*. Tm evasion.

## Discussion

Earlier work has implicated NAIP/NLRC4 in host defense against *S*. Tm and related enteropathogens.^8,10,11,30,44,47,55^ Mice globally lacking key components of this inflammasome suffer from exacerbated systemic infection and higher pathogen loads in lymph nodes, spleen and liver upon oral inoculation.^8,10,11,37,44,47^ As mechanistic work on NAIP/NLRC4 signaling has focused on phagocytic cell types,^26^ and as *S*. Tm frequently lodge within such cells at extraintestinal locations,^58^ it has been assumed that phagocytes mediate protection of systemic organs via NAIP/NLRC4. In sharp contrast, our analysis proves phagocyte NAIP/NLRC4 to be dispensable for restriction of *S*. Tm migration to, and replication at, systemic sites (Figs. 1, 3, 4, S5A) during early infection. This pertains also to an *S*. Tm strain that enters promptly into lamina propria phagocytes upon passive uptake of luminal bacteria (*S*. Tm^*Δ4*^; Fig. 3b, c). Instead, our data show that inflammasome defense against dissemination of different *S*. Tm strains from the gut relies specifically on intestinal epithelial NAIP/NLRC4 (Fig. 3, S5), previously shown to drive the expulsion of infected IECs and restrict mucosal pathogen loads by >>50-fold during oral *S*. Tm or *Citrobacter rodentium* infection.^11,55^ These results establish IEC NAIP/NLRC4 as the warden of not only the gut mucosa, but also of the systemic compartment, upon infection with a host-adapted pathogen.

The expression levels of *Naip* and *Nlrc4* transcripts are notably high in IECs, but also clearly detectable across systemic organs and mucosal tissue DCs (Fig. 5, Fig. S6). On top of this, the downregulation of NAIP-activating PAMPs within host tissues provides an explanation to the failure of phagocytes, but ability of IECs, to utilize NAIP/NLRC4 for combatting *S*. Tm in vivo (Fig. 6, Fig S7–S8).

Following flagella- and TTSS-1-driven invasion of IECs, which by necessity exposes these PAMPs to epithelial NAIP receptors, *S*. Tm promptly downregulates expression of both structures, thereby preventing NAIP/NLRC4 activation beyond the epithelial barrier (Fig. 6, Fig S8–S9).^93^ Other evasion mechanisms, e.g., SCV membrane shielding of PAMPs from the cytosolic NAIPs, and active interference by secreted virulence factors, might further dampen residual inflammasome activation in a cell-type specific manner.^1,24^ Also, it remains to be fully shown how polymerization into the flagellum affects the NAIP/NLRC4 triggering intensity of flagellin. Anyhow, the evasion of recognition is possibly already employed during IEC-invasion, but may be hampered by high bacterial invasion rates and relatively long lifetimes of the bacterial proteins. Nevertheless, even when administered systemically, *S*. Tm is not recognized by phagocyte NAIP/NLRC4, except if expression of the flagella component FliC, a NAIP5-6 ligand, is artificially forced (Fig. 4, Fig S9).^92^ Therefore, while phagocytes at systemic sites would indeed be capable of pathogen restriction via NAIP/NLRC4 (Fig. 5, Fig S9), our population dynamics data conclusively show that different *S*. Tm strains evade this defense. This is in stark contrast to less-well adapted bacteria, like *Chromobacterium violaceum*, which are vigorously restricted (>>1.000-fold in liver) by the NAIP/NLRC4 inflammasome in systemic organs.^94^ Based on these observations, it is tempting to speculate that mechanisms for avoiding the NAIP/NLRC4 defense have contributed to the evolution of gene-regulatory circuits in host-adapted bacterial pathogens.

Other inflammasomes have previously been deemed protective during *S*. Tm infection in mice.^37,39,43^ However, under stringent littermate-controlled conditions, we here find that neither NLRP3, nor Caspase-11, impact *S*. Tm transmucosal migration or early systemic replication in vivo, at least in the Streptomycin pretreated mouse model (Fig. 2). Caspase-11 can protect against systemic infection by bacteria colonizing the host cell's cytosol, e.g., *Burkholderia* spp.^40^ Other intracellular pathogens, including *S*. Tm and *Legionella pneumophila* (*L. pn*), express virulence factors that stabilize the intracellular vacuole.^88,95^ Mutant *S*. Tm and *L. pn* strains lacking those factors breach the vacuolar membrane with higher frequency and are efficiently detected by Caspase-11.^40,42^ Our finding that Caspase-11 is dispensable for restriction of *S*. Tm dissemination/replication (Fig. 2, Fig S3) agrees with these reports, supporting that wildtype *S*. Tm evades Caspase-11 recognition after oral infection in vivo. Importantly, deletion of *Casp11* in *Nlrc4*^−/−^ mice does not increase pathogen migration or systemic colonization (Figs. 2, S3, 4). This refutes the possibility that a potent epithelial NAIP/NLRC4 response masks any effect of Caspase-11. Furthermore, *Casp1/11*^−*/*−^ animals only partially recapitulate the elevated *S*. Tm dissemination of NAIP1-6 or NLRC4-deficient animals (Figs. 2, S3, 4). This points to the potential involvement of also other Caspases, e.g., Caspase-8,^10^ in the execution of the IEC NAIP/NLRC4 response. The molecular wiring of this defense system will be an important topic for future work.

During acute infection, NLRP3 does not contribute to restriction of *S*. Tm infection neither in the gut mucosa (Fig. 2, Fig S3), nor at systemic sites (Fig. 4), not even in the absence of NLRC4 (Figs. 2, S3, 4). This contrasts to previous reports in the literature.^37,39^ We can only speculate about the origins of this discrepancy. Our results apply to different, widely used *S*. Tm strains (SL1344 and 14028), rendering the use of different strains an unlikely cause. It might be attributable to the huge impact of the intestinal microbiota on non-typhoidal *Salmonella* infection models, as well as to small differences in genetic backgrounds that have recently been revealed.^51–53^ These could have been confounding in experiments using separately bred wildtype mice as controls rather than littermates. Again, the lack of an NLRP3-linked response may also be explained by efficient *S*. Tm evasion of this inflammasome.^96^ Furthermore, NLRP3 and/or Caspase-11 may serve some more specific function(s) during late stages of a persistent infection or in hosts other than mice.

Taken together, our data extend previous work,^10,11,37,39,42,44,45,47,49,50,76^ highlight that in vivo studies of host–pathogen interactions are highly sensitive to the experimental conditions, and may be subject to confounding effects that limit reproducibility. The general approach presented here—a combination of littermate controlled infections, host cell type-specific gene knockouts, genetically tagged bacterial consortia, mathematical modeling and high-resolution imaging of the infected cells/organs—allowed us to decipher the impact of specific host responses in vivo. Here, this led us to refute a protective function for phagocyte inflammasome defense and to uncover intestinal epithelial NAIP/NLRC4 as a firewall preventing systemic dissemination of the orally acquired adapted pathogen *S*. Tm. We expect the same approach to be powerful for (re-) assessing also the impact of other barriers during the step-wise progression of infectious disease, with extension to other pathogens.

## Methods

### *Salmonella* strains and growth conditions

Strains used in this study are *S*. Tm SL1344 (SB300, Streptomycin resistant), or derivatives thereof, except for *S*. Tm^14028^ ^97^ and WITS^14028^. *S*. Tm^Δ4^ lacks four TTSS-1 effector proteins (*sipA*, *sopB*, *sopE*, *sopE2*), but still expresses the TTSS-1 secretion apparatus, and was described previously.^71^ The tags for the WITS strains as described in ref.,^58^ were introduced by P22 phage transduction. We used *S*. Tm carrying pM975 (*S*. Tm^SPI-2-GFP^)^72^ as a reporter for SPI-2 expression (*S*. Tm,^SPI-2^ within the SCV), *S*. Tm expressing GFP under a *prgH*-promotor (JH3010, *S*. Tm^SPI-1-GFP^) to mark SPI-1 expressing bacteria (*S*. Tm^SPI-1^), *S*. Tm carrying pCK100 (Glc6P-driven mCherry expression, *S*. Tm^Glc6P-mCherry^) as a reporter for cytosolic bacteria (*S*. Tm^Glc6P^) and *S*. Tm^localizer^ expressing GFP under the *ssaG* promotor (JH3009) and carrying pCK100 to track SCV/cytosolic localization of *S*. Tm. pCK100 was constructed as follows: A 137 bp genomic fragment immediately upstream of the *uhpT* gene in *Shigella flexneri* 2a 2457T (GenBank: AE014073.1, 3869168...3869032) was cloned upstream of the ribosomal binding site of phage T7 gene 10 and mCherry on a pSC101 backbone. *S*. Tm carrying this plasmid showed induced red fluorescence when cultured in presence of Glc6P but not in presence of glucose. As Glc6P is specifically present in the host cell cytosol, this is a reporter plasmid for cytosolic localization, as described previously for *Shigella flexneri*.^98^ *S*. Tm^*ΔfliGHI*^ carrying pM975 (*pssa*G-GFPmut2) was used as a control for specificity of the FliC staining. *S*. Tm^*fliCind*^ was generated by electroporation of the plasmid pEM087^14^ into *S*. Tm.^Δ*flgB*^ *S*. Tm^Δ*flgB*^ carrying an empty control plasmid was used as “wildtype” control in the respective experiment (*S*. Tm,^ECV^ Fig S7). Genetic barcodes were introduced by P22 phage transduction into *S*. Tm^*fliCind*^ and *S*. Tm^ECV^ for relative quantification of the abundance of the two strains during competitive infection. See Table 1 for further information. For oral and HeLa cell infections, *S*. Tm was grown overnight at 37 °C in LB/0.3M NaCl and sucultured for four hours at a dilution of 1:20. For iv infections, *S*. Tm was grown overnight in LB/0.3M NaCl.

**Table 1:** Strains used in this study.

Strain name in manuscript	Strain number	Relevant genotypes	Comment	Resistances	Reference
SL1344	SB300	wildtype	Referred to as *S*. Tm	Sm	^106^
SB300^TAG1^	M3147	*Tag1-aphT*	Collectively referred to as WITS	Sm, Km	^59^
SB300^TAG2^	M3148	*Tag2-aphT*		Sm, Km	
SB300^TAG11^	M3149	*Tag11-aphT*		Sm, Km	
SB300^TAG13^	M3150	*Tag13-aphT*		Sm, Km	
SB300^TAG17^	M3151	*Tag17-aphT*		Sm, Km	
SB300^TAG19^	M3152	*Tag19-aphT*		Sm, Km	
SB300^TAG21^	M3153	*Tag21-aphT*		Sm, Km	
*S*. Tm^Δ4^	M566	*ΔsipAsopBsopEsopE2*	IEC invasion-deficient mutant	Sm	^71^
*S*. Tm^Δ4 TAG1^	Z2557	*ΔsipAsopBsopEsopE2, Tag1-aphT*	Collectively referred to as WITS^Δ4^	Sm, Km	This study
*S*. Tm^Δ4 TAG2^	Z2558	*ΔsipAsopBsopEsopE2, Tag2-aphT*		Sm, Km	This study
*S*. Tm^Δ4 TAG11^	Z2559	*ΔsipAsopBsopEsopE2, Tag11-aphT*		Sm, Km	This study
*S*. Tm^Δ4 TAG13^	Z2560	*ΔsipAsopBsopEsopE2, Tag13-aphT*		Sm, Km	This study
*S*. Tm^Δ4 TAG17^	Z2561	*ΔsipAsopBsopEsopE2, Tag17-aphT*		Sm, Km	This study
*S*. Tm^Δ4 TAG19^	Z2562	*ΔsipAsopBsopEsopE2, Tag19-aphT*		Sm, Km	This study
*S*. Tm^Δ4 TAG21^	Z2563	*ΔsipAsopBsopEsopE2, Tag21-aphT*		Sm, Km	This study
*S*. Tm^SPI-2-GFP^	SB300	p*ssaG-GFPmut2*	p*ssaG-GFPmut2* (pM975): expression of GFPmut2 under *ssaG* (SPI-2) promotor, which is active inside the SCV (*S*. Tm^SPI-2^)	Sm, Amp	^72^
*S*. Tm^SPI-1-GFP^	Z1404	*prgH-GFP*	SPI-1 regulated expression of GFP (*S*. Tm^SPI-1^)	Sm, Cm	^107^
*S*. Tm^localizer^	Z1440	*ssaG*-*GFP* p*uhpT-mCherry*	p*uhpT-mCherry* (pCK100/pZ1400): Glc6P induced mCherry expression as a reporter for cytosolic localization (*S*. Tm^Glc6P^). In addition, this strain contains an *ssaG*-driven GFP reporter for SCV localization (*S*. Tm^SPI-2^)	Sm, Amp, Cm	This study
*S*. Tm^Glc6p-mCherry^	Z1439	p*uhpT-mCherry*	p*uhpT-mCherry* (pCK100/pZ1400): Glc6P induced mCherry expression as a reporter for cytosolic localization (*S*. Tm^Glc6P^).	Sm, Amp	This study
*S*. Tm^Δ*fliGHI*^	M913	*ΔfliGHI* p*ssaG-GFPmut2*	Flagella-deficient mutant. Also contains p*ssaG-GFPmut2* (pM975): expression of GFPmut2 under *ssaG* (SPI-2) promotor, which is active inside the SCV (*S*. Tm^SPI-2^)	Sm, Amp, Tet	^108^
*S*. Tm^*fliCind*^	Z2500	Δ*flgB, Tag1-aphT* pEM087	Flagella-deficient mutant. Also contains pEM087: Doxycycline inducible expression of FliC	Sm, Cm, Amp, Tet, Km	^14^
*S*. Tm^ECV^	Z2530	Δ*flgB, Tag13-aphT* pZ2500	Flagella-deficient mutant. Also contains pZ2500: Empty control vector for pEM087 carrying a Tet (Doxycycline) resistance cassette	Sm, Cm, Amp, Tet, Km	^14^
14028	M3168	*IpfED::aphT*	Referred to as *S*. Tm^14028^	Km	^97^
14028^TAG1^	Z6708	*IpfED::aphT*, *Tag1-cat*	Collectively referred to as WITS^14028^	Km, Cm	This study
14028^TAG2^	Z6709	*IpfED::aphT*, *Tag2-cat*		Km, Cm	This study
14028^TAG11^	Z6710	*IpfED::aphT*, *Tag11-cat*		Km, Cm	This study
14028^TAG13^	Z6711	*IpfED::aphT*, *Tag13-cat*		Km, Cm	This study
14028^TAG17^	Z6712	*IpfED::aphT*, *Tag17-cat*		Km, Cm	This study
14028^TAG19^	Z6713	*IpfED::aphT*, *Tag19-cat*		Km, Cm	This study
14028^TAG21^	Z6714	*IpfED::aphT*, *Tag21-cat*		Km, Cm	This study

Sm = Streptomycin. Km = Kanamycin. Cm = Chloramphenicol. Amp = Ampicillin. Tet = Tetracycline.

### Tissue culture and infections

HeLa CCL-2 cells (ATCC) were grown in DMEM (Gibco) supplemented with 10% inactivated FCS (Thermo Fischer) and 50 μg/mL Streptomycin (AppliChem) at 37 °C and 5% CO_2_. For immunofluorescence experiments, 80.000 HeLa cells were seeded in 24-well plates (Nunc, Thermo Fisher) containing glass cover slips 24 h prior to infection. Cells were infected with *S*. Tm^SPI-2-GFP^ at an estimated MOI of 300, *S*. Tm^localizer^ at an estimated MOI of 300, and *S*. Tm^Δ*fliGHI*^ (p*ssaG*-GFPmut2) at an estimated MOI of 1000 for 14 mpi, 1 hpi, 3 hpi, 5 hpi, and 7 hpi (*S*. Tm,^SPI-2-GFP^ *S*. Tm^localizer^) and 14 mpi and 1 hpi (*S*. Tm^Δ*fliGHI*^(p*ssaG*-GFPmut2)). After 20 min, infected HeLa cells were washed three times with DMEM/10% FCS and incubated with DMEM/10% FCS containing 400 μg/mL Gentamicin to prevent further infection. For electron microscopy (EM) imaging experiments, EM finder grids (gold NH2 R2/2, Quantifoil) were sterilized under UV light and then glow discharged. Grids were placed on the bottom of the wells of a 12-well plate (Nunc, Thermo Fisher) and equilibrated with DMEM for 30 min. Subsequently, 30,000 HeLa cells were seeded into each well (containing one grid each) and incubated overnight. Cells were infected with *S*. Tm^SPI-2-GFP^ at an estimated MOI of 300 for 1 h as described above. Grids were washed twice with HBSS before vitrification.

### Mice and infections

All studies were performed in accordance with ethical and legal requirements and were approved by the Kantonales Veterinäramt Zürich under the licenses 222/2013 and 193/2016. Mice were kept under specific pathogen-free conditions in individually ventilated cages (EPIC and RCHCI, ETH Zürich). All knockout mouse lines presented here have a C57BL/6 background, *Naip1-6*,^−*/*−^ *Naip1-6*^*ΔIEC*^, and *Naip1-6*^*ΔCD11c*^ have a C57BL/6J background, *Nlrc4*^−/−^ mice a C57BL/6NJ background. *S*. Tm infections were performed as described before^56^ on 8–12 week old mice. Cohoused, heterozygous littermates were used as controls in all infections. Control mice for Fig. 8B were supplemented with unrelated wild type mice (*Nlrc4*^+/+^). For oral infection, mice were pretreated orally with 25 mg Streptomycin or 10 mg Kanamycin (for *S*. Tm^14028^) 24 h prior to infection. Infection was performed by intragastrical inoculation with 5 × 10^7^ bacteria in 50 µl PBS. For WITS infections, the WITS strains (Kanamycin resistant) were diluted 1:140 (for wildtype *S*. Tm) or 1:21 (for *S*. Tm^*Δ4*^) in the respective untagged *S*. Tm strain. For iv infection, 10^4^ *S*. Tm in 100 µl PBS were injected into the tail vein. For WITS iv infections, WITS strains (Kanamycin resistant) were diluted 1:700 in untagged wildtype *S*. Tm. The infection with *S*. Tm^*fliCind*^ was performed by iv injection of 10^4^ CFU in a 1:1 mix of *S*. Tm^*fliCind*^ and *S*. Tm.^ECV^ Both strains showed equal growth in vitro (data not shown). At 17 hpi, 0.8 mg Doxycycline in 100 µl PBS was administered iv for induction of FliC expression. At 24 hpi, mice were euthanized and organs and cecal content were collected, homogenized, and plated on MacConkey Agar (Oxoid) containing the respective antibiotics for enumeration of bacterial counts in the respective tissue. For competitive infections, relative abundance of the two competitors in the tissue of interest was assessed by quantitative real-time PCR (qPCR) with the help of neutral genetic tags as described below.

### LPS injections and sorting of intestinal DCs

Mice were iv injected with 5 μg ultrapure *S*. Tm LPS (Otto Holst) in 100 μl PBS, or 100 μl PBS as control. One hour post injection, mice were euthanized and cecae were excised. Cecum tissue was washed extensively with PBS, cut into small pieces and incubated twice for 20 min at 37 °C shaking in 13 ml PBS supplemented with 5 mM EDTA (Life Technologies), 15 mM HEPES (Life Technologies) and 10% heat-inactivated FBS (Life Technologies). Tissue pieces were washed in 7 ml RPMI 1640 (Life Technologies)/30% FBS and subsequently incubated for 1 h at 37 °C shaking in 900 μl RPMI containing 0.2 mg/ml DNase I (Roche) and 1 mg/ml collagenase VIII (Sigma). Digested material was mashed through a 70 μm cell strainer and washed with 10 ml RPMI. After washing, cells were resuspended in 6 ml RPMI, carefully loaded onto 3 ml NycoPrep 1.077 and centrifuged for 30 min at 400 × *g* (room temperature). The interphase fraction was collected (~2 ml), washed in 6 ml RPMI containing 15% FBS, and subsequently stained for fluorescent activated cell sorting (FACS). Cells from three mice were pooled per sample for sorting. The following antibodies were used for staining of intestinal DCs: CD45-PerCP (Biolegend, 30-F11, 1:100), MHCII-APC (Biolegend, M5/114.15.2, 1:400), CD103-PE (Biolegend, 2E7, 1:100), CD11b-BV605 (Biolegend, M1/70, 1:200), CD11c-PE/Cy7 (Biolegend, N418, 1:200), Sytox-blue (Invitrogen, 1:1000). Intestinal DCs were sorted for CD45^+^ MHCII^hi^ CD11c^hi^ live cells and differentiated into CD103^+^ CD11b,^−^ CD103^−^ CD11b,^+^ CD103^+^ CD11b^+^, and CD103^−^ CD11b^−^ populations. Cells were FACS sorted with a BD FACSAria III sorter into 50 μl RNAlater (Sigma) and flash frozen in liquid nitrogen.

### WITS quantification

WITS-tagged *S*. Tm from mLN (oral infection) or spleen (iv infection) homogenate were specifically enriched in 3 ml LB supplemented with 50 µg/ml Kanamycin overnight. Bacterial DNA was isolated from enrichment cultures (Qiagen QIAamp DNA Mini Kit) and subsequently analyzed on a StepOne Plus Cycler (Thermo Fisher), using FastStart Universal SYBR Green Master (Rox) reagents (Roche) and primers as described before.^58^ Total abundance of the WITS strains was assessed by integrating their relative distribution analyzed by qPCR with total bacterial CFU obtained through plating.

### Population dynamics analysis

Population dynamics were analyzed with a previously published model,^59^ based on total WITS counts in the organ of interest of each mouse. This method was developed for the estimation of the migration rate *µ* from the gut lumen to the mLN and the replication rate *r* within the mLN during oral *S*. Tm infection, and was directly used for the estimates of the oral infection experiments (Figs. 1–3, S5). We applied the same method to estimate the migration rate *µ* from the blood to and the replication rate *r* within the spleen for the iv infection experiments (Fig. 4). We note that the interpretation of the migration rate estimate for this application of the model differs from the original application. The migration is assumed to be constant over time in the model, however, previous research has shown that the number of bacteria in the blood exponentially decreases for iv inoculation.^99^ Therefore, the migration we estimate to occur with a constant rate over one day, might have migrated only within the first few hours after inoculation. However, because we are not primarily interested in the migration rates themselves but rather the differences in migration rates between wildtype and knockout mice, this interpretational subtlety does not confound our conclusions. Calculations were performed with R Studio, R version 3.6.0.

### Gene expression analysis

For gene expression analysis, tissue was snap frozen in RNAlater (Sigma-Aldrich). RNA isolation was performed with the Qiagen RNeasy Mini Kit according to the manufacturer's instructions, including DNase digestion. For sorted intestinal DCs, RNA isolation was performed with the Qiagen RNeasy Micro Kit after cell lysis with the QIAShredder Kit (Qiagen) according to the manufacturer`s instructions. One microgram of isolated RNA was subsequently transcribed into cDNA using the Qiagen RT2 HT First Strand cDNA Kit, and stored at −20 °C until analysis. qPCR was performed with FastStart Universal SYBR Green Master (Rox) reagents (Roche) on a StepOne Plus Cycler (Thermo Fischer). mRNA levels were normalized to *Actb* and calculated with the 2^−ΔCT^-method. Primers for the indicated genes were purchased from Qiagen (RT2 qPCR Primer Assay).

### Histopathology

For the assessment of histopathology, cecal tissue was snap-frozen in OCT (Tissue-Tek) and stored at −80 °C. Five micrometer sections were cut from the tissue and stained with hematoxylin and eosin as described before.^56^ Tissue pathology was scored according to the extent of submucosal edema, epithelial integrity, goblet cell loss and infiltration of polymorphonuclear neutrophils (PMNs) into the lamina propria.

### Fluorescence microscopy

Infected HeLa cells were fixed with 4% Paraformaldehyde (Sigma-Aldrich) for 15 min (room temperature) and subsequently permeabilized with 0.1% Triton X-100 for 5 min (room temperature). Afterwards cells were washed twice with 4% sucrose and incubated in 20% sucrose for 20 min. Next, cells were incubated with blocking buffer for 1 h (3% bovine serum albumin, BSA; 3% sucrose in DPBS) before staining with the respective primary antibodies (rabbit-αLPS, Difco^TM^ *Salmonella* O Antiserum, 1:200; mouse-αFliC, Abcam, 1:300) for 1 h. After three washes with PBS samples were incubated with secondary antibodies (goat-αmouse IgG Cy5; goat-αrabbit IgG Alexa Fluor 405; 1:600, Thermo Fischer) for 1h. Finally, samples were washed three times with PBS and mounted on microscope slides with 5 μl Mowiol. For immunofluorescence staining of infected organs, cecal tissue and spleens of infected mice were fixed in 4% Paraformaldehyde (Sigma-Aldrich) for 4 h at 4 °C, dehydrated in 20% sucrose for 4 h at 4 °C, embedded in OCT (Tissue-Tek), flash frozen and stored at −80 °C. For detection of flagellated intracellular *S*. Tm, 20 µm sections of cecal mucosa and spleen were prepared. After rehydration (PBS, 1 min), sections were permeabilized with 0.5% Triton X-100 for 5 min (room temperature). Next, samples were incubated in blocking buffer (10% normal goat serum, Reactolab) for 30 min. Sections from *JH*^−/−^ mice were stained for 40 min (room temperature) using a mouse-αFliC antibody (Abcam, 1:300). Sections from *Naip1-6*^*Δ/ΔIEC*^ knockout mice were incubated with a FliC-Cy3-conjugated antibody (1:300; Cy3^®^ Fast Conjugation Kit Abcam; ab188287). After three washes with PBS, samples were stained for 40 min (αmouse IgG-Cy3, 1:200; Phalloidin-A647, 1:200; DAPI, 1:1000; Thermo Fischer). Finally, samples were washed three times with PBS and a coverslip was mounted on the microscope slide with 15 μL Mowiol. Image acquisition was performed with a Nikon Eclipse T1 (inverse) microscope equipped with a Yokogawa CSU-W1-T2 spinning-disk confocal unit (Visitron), a sCMOS camera (Orca Flash 4.0 V2) and a ×100 oil objective (PLAN Apochromat, NA 1.49). All data were analyzed in Fiji.^100^

### Preparation of frozen-hydrated specimens

Plunge freezing was performed as previously described.^101^ Briefly, grids containing infected HeLa cells, were removed from the wells using tweezers. The forceps were then mounted in the Vitrobot chamber and the grid was blotted from the backside by installing a Teflon sheet on one of the blotting pads. Grids were plunge-frozen in liquid ethane-propane (37%/63%) using a Vitrobot (Thermo Fisher) and stored in liquid nitrogen.^102^

### Cryo-focused ion beam milling

Cryo-focused ion beam (cryoFIB) milling was used to prepare samples of plunge-frozen infected HeLa cells that could then be imaged by cryo-electron tomography.^103^ Frozen grids with infected HeLa cells were prepared and processed as previously described.^102^ Briefly, lamellae were milled in several steps in a Helios NanoLab600i dual beam FIB/SEM instrument (Thermo Fisher). In a first step, two rectangular regions were used to generate a lamella with ~2 µm thickness with the ion beam set to 30 kV and ~400 pA. The current of the ion beam was then gradually reduced until the lamella reached a nominal thickness of ~250 nm (ion beam set to ~25 pA). The stage temperature was maintained below −154 °C during loading, milling and unloading procedures. CryoFIB-processed grids were unloaded and stored in liquid nitrogen until further use.

### Cryo-electron microscopy and cryo-electron tomography

CryoFIB processed infected HeLa cells were examined by cryo-electron microscopy (cryoEM) and cryo-electron tomography (cryoET).^101,102^ Images were recorded on a Titan Krios TEM (Thermo Fisher) equipped with a Quantum LS imaging filter and K2 Summit (Gatan). The microscope was operated at 300kV and the imaging filter was set to a 20 eV slit width. The pixel size at the specimen level was 5.42 Å. Tilt series covered an angular range from −60° to +60° with 2° increments and −8 µm defocus. The total dose of a tilt series was 120 e^−^/Å.^2^ Tilt series and 2D projection images were acquired automatically using SerialEM.^104^ Three-dimensional reconstructions and segmentations were generated using the IMOD program suite.^105^

## Supplementary information

Supplementary Inormation
