# Supplementary material for: Intestinal epithelial NAIP/NLRC4 restricts systemic dissemination of the adapted pathogen Salmonella Typhimurium due to site-specific bacterial PAMP expression
Source: Mucosal Immunol. 2020 Jan 17;13(3):530–44. doi: 10.1038/s41385-019-0247-0 (PMC7181392; doi:10.1038/s41385-019-0247-0)
Supplement: Supplementary file 1 — Supplementary Inormation [file 41385_2019_247_MOESM1_ESM.pdf]

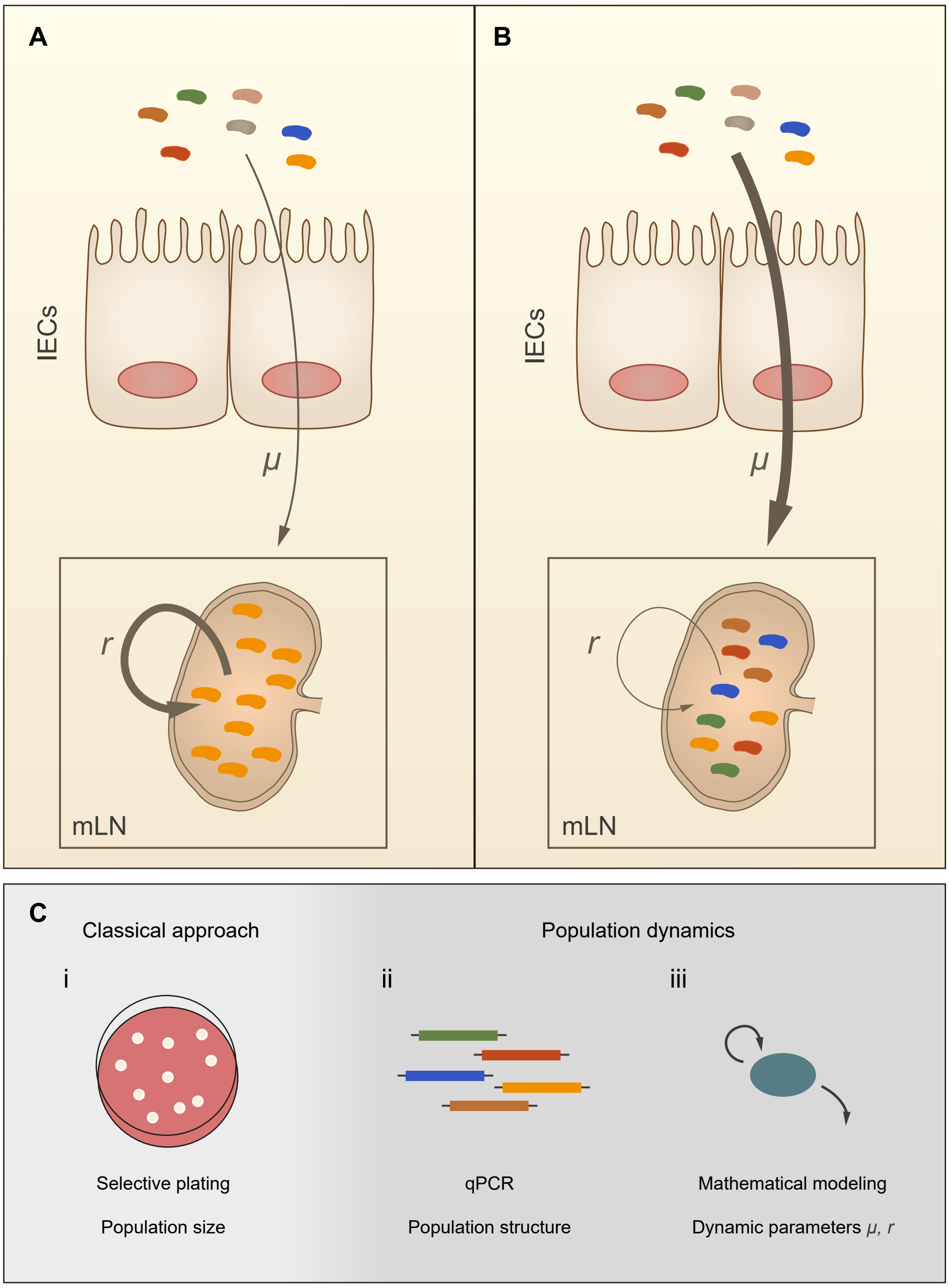

A
IECs
μ
r
mLN
B
IECs
μ
r
mLN
C
Classical approach
Population dynamics
i
ii
iii
Selective plating
Population size
qPCR
Population structure
Mathematical modeling
Dynamic parameters μ, r

Figure S1

Figure S1: Schematic overview of the experimental approach: The use of genetically tagged *S*. Tm for the quantitation of population dynamics during oral infection. Scenario **A** (low migration rate (μ), high net replication rate (r, integration of replication and clearance within the mLN)) and scenario **B** (high migration rate (μ) and low net replication in the mLN (r)) can result in the same pathogen loads in the mLN, and can therefore not be differentiated by conventional selective plating as shown in **C** (classical approach). Notably, using tagged WITS strains (represented by the different colors), analysis of tag-distribution within the mLN by qPCR provides information of the population structure and makes differentiation of the two scenarios possible. Mathematical modeling allows precise quantification of the contribution of the dynamic parameters μ and r to the total pathogen population within the mLN.

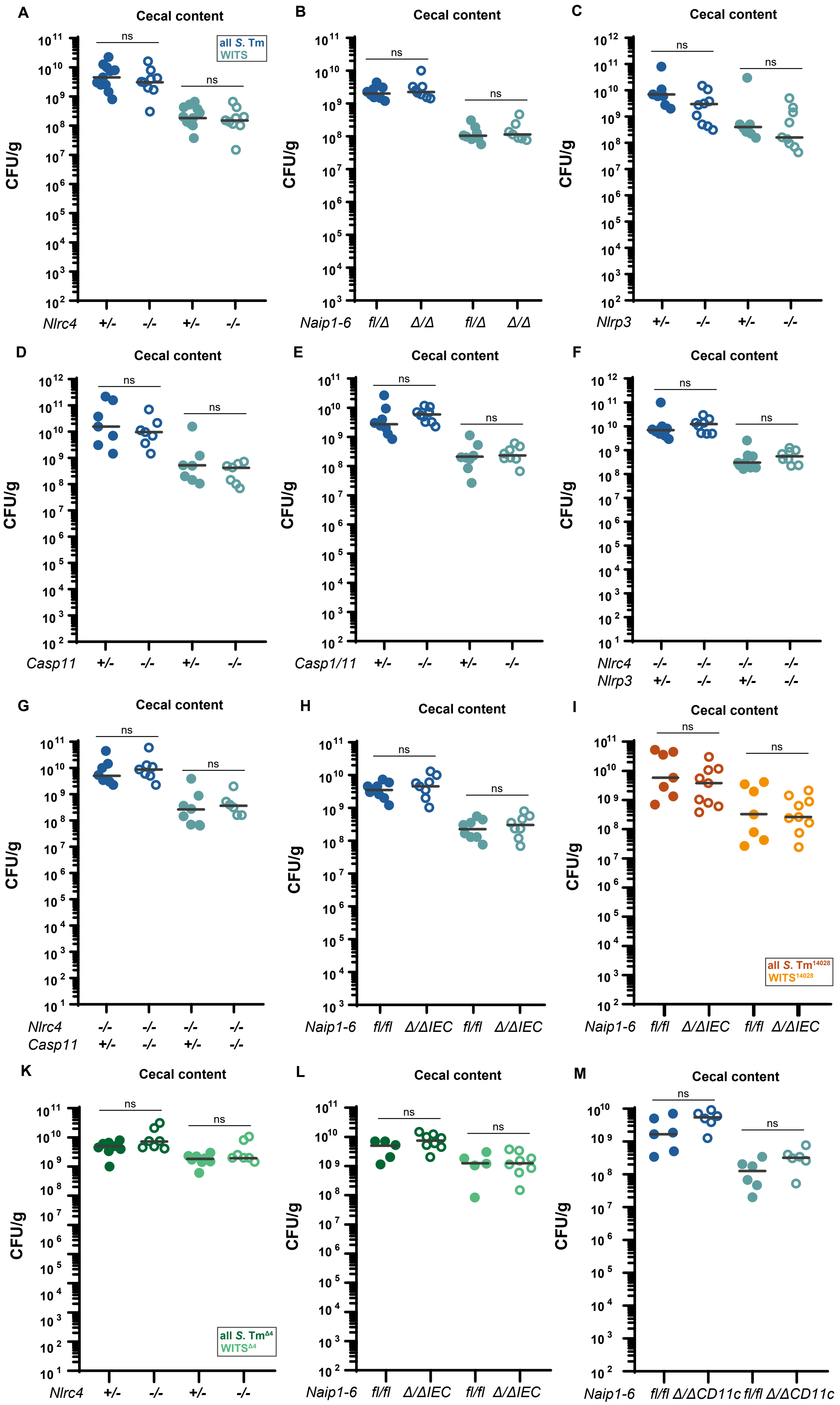
A
Cecal content
all S. Tm
WITS
CFU/g
Nlrc4 +/- -/- +/- -/-
B
Cecal content
CFU/g
Naip1-6 fl/Δ Δ/Δ fl/Δ Δ/Δ
C
Cecal content
CFU/g
Nlrp3 +/- -/- +/- -/-
D
Cecal content
CFU/g
Casp11 +/- -/- +/- -/-
E
Cecal content
CFU/g
Casp1/11 +/- -/- +/- -/-
F
Cecal content
CFU/g
Nlrc4 -/- -/- -/- -/-
Nlrp3 +/- -/- +/- -/-
G
Cecal content
CFU/g
Nlrc4 -/- -/- -/- -/-
Casp11 +/- -/- +/- -/-
H
Cecal content
CFU/g
Naip1-6 fl/fl Δ/ΔIEC fl/fl Δ/ΔIEC
I
Cecal content
CFU/g
all S. Tm14028
WITS14028
Naip1-6 fl/fl Δ/ΔIEC fl/fl Δ/ΔIEC
K
Cecal content
CFU/g
all S. TmΔ4
WITSΔ4
Nlrc4 +/- -/- +/- -/-
L
Cecal content
CFU/g
Naip1-6 fl/fl Δ/ΔIEC fl/fl Δ/ΔIEC
M
Cecal content
CFU/g
Naip1-6 fl/fl Δ/ΔCD11c fl/fl Δ/ΔCD11c

Figure S2

Figure S2: Deletion of inflammasome components does not alter luminal *S*. Tm colonization. *S*. Tm cecal luminal counts (CFU/gram cecal content) in **A** *Nlrc4*$^{+/-}$ (circles) and *Nlrc4*$^{-/-}$ (open circles) mice shown in Figure 1A **B** *Naip1-6*$^{\Delta/fl}$ (circles) and *Naip1-6*$^{\Delta/\Delta}$ (open circles) mice shown in Figure 1E **C** *Nlrp3*$^{+/-}$ (circles) and *Nlrp3*$^{-/-}$ (open circles) mice shown in Figure 2A-B and S3A **D** *Casp11*$^{+/-}$ (circles) and *Casp11*$^{-/-}$ (open circles) mice shown in Figure 2A-B and S3B **E** *Casp1/11*$^{+/-}$ (circles) and *Casp1/11*$^{-/-}$ (open circles) mice shown in Figure 2A-B and S3C **F** *Nlrc4*$^{-/-}$*Nlrp3*$^{+/-}$ (circles) and *Nlrc4*$^{-/-}$*Nlrp3*$^{-/-}$ (open circles) mice shown in Figure 2C-D and S3D **G** *Nlrc4*$^{-/-}$*Casp11*$^{+/-}$ (circles) and *Nlrc4*$^{-/-}$*Casp11*$^{-/-}$ (open circles) mice shown in Figure 2C-D and S3E **H** *Naip1-6*$^{fl/fl}$ (circles) and *Naip1-6*$^{\Delta/\Delta IEC}$ (open circles) mice shown in Figure 3A **I** *Nlrc4*$^{+/-}$ (circles) and *Nlrc4*$^{-/-}$ (open circles) mice infected with *S*. Tm$^{\Delta4}$ shown in Figure 3B **K** *Naip1-6*$^{fl/fl}$ (circles) and *Naip1-6*$^{\Delta/\Delta IEC}$ (open circles) mice infected with *S*. Tm$^{\Delta4}$ shown in Figure 3C **L** *Naip1-6*$^{fl/fl}$ (circles) and *Naip1-6*$^{\Delta/\Delta IEC}$ (open circles) mice infected with *S*. Tm^{14028} shown in Figure S5A **M** *Naip1-6*$^{fl/fl}$ (circles) and *Naip1-6*$^{\Delta/\Delta CD11c}$ (open circles) mice shown in Figure S5B. Depicted are counts of all *S*. Tm (dark blue, selected for with Streptomycin, green for *S*. Tm$^{\Delta4}$, selected for with Streptomycin; brown *S*. Tm^{14028}, selected for with Kanamycin) and specifically of the WITS (light blue for WITS, light green for WITS$^{\Delta4}$, selected for with Kanamycin; orange for WITS^{14028}, selected for with Chloramphenicol). Each circle represents one mouse. Combined data of at least three independent experiments (see respective main figures). Grey line: Median. Statistical analysis: Mann-Whitney-U Test, p-values indicated, ns: $p \geq 0.05$.

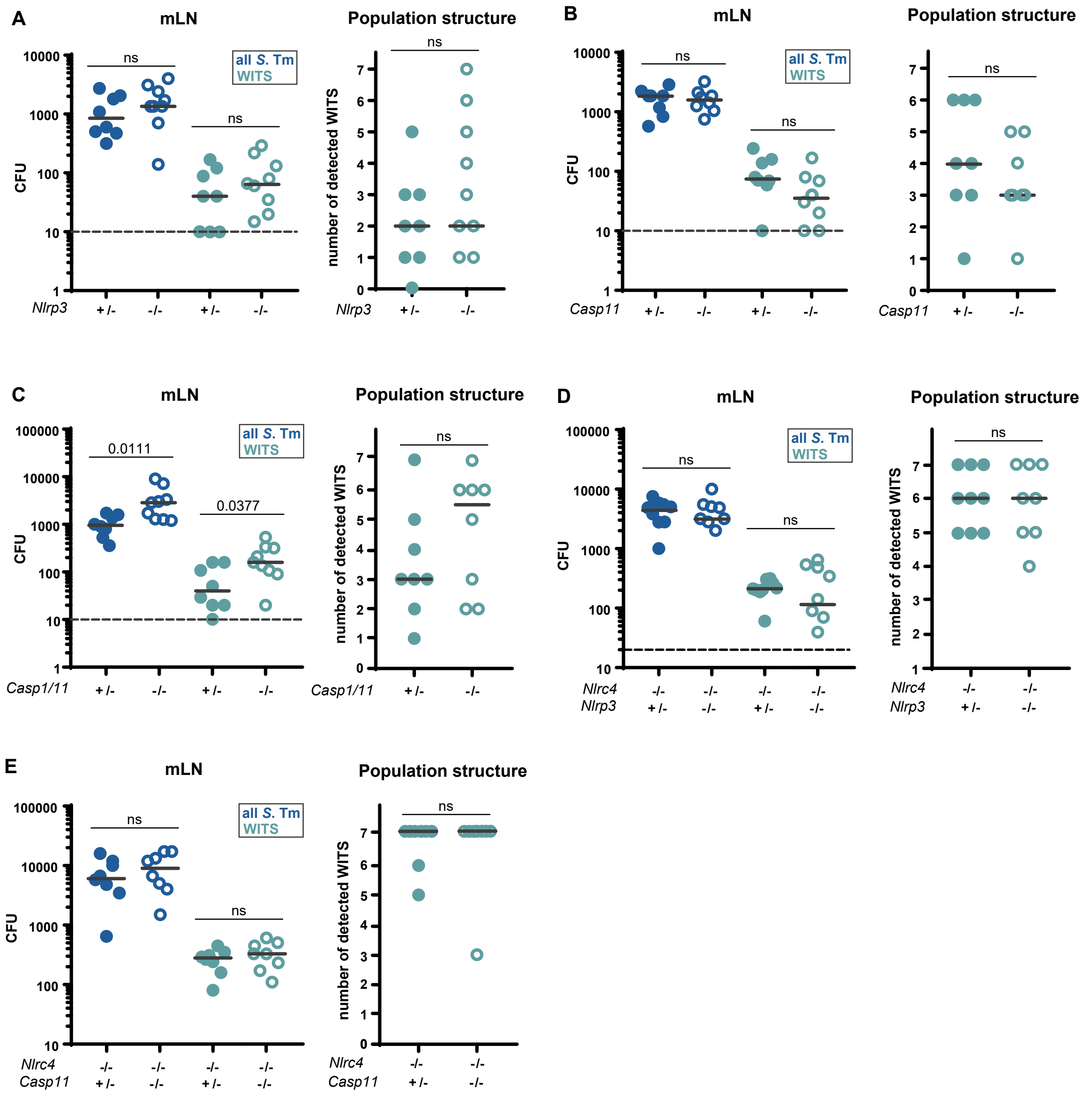
A
mLN
Population structure
B
C
D
E
all S. Tm
WITS
CFU
number of detected WITS
ns
0.0111
0.0377
Nlrp3
Casp11
Casp1/11
Nlrc4
+/-
-/-

Figure S3

Figure S3: NLRP3 and Caspase-11 do not contribute to restriction of systemic *S*. Tm spread during oral infection, while Caspase-1 is partially involved. Streptomycin pretreated mice were orally infected with $5x10^7$ CFU *S*. Tm. *S*. Tm counts in the mLN at 24 hpi are independent of NLRP3 (**A**, *Nlrp3*$^{+/-}$ (circles) and *Nlrp3*$^{-/-}$ (open circles)) and Caspase-11 (**B**, *Casp11*$^{+/-}$ (circles) and *Casp11*$^{-/-}$ (open circles)). Caspase-1 partially contributed to restriction of mLN loads (**C**, *Casp1/11*$^{+/-}$ (circles) and *Casp1/11*$^{-/-}$ (open circles)). Even in absence of NLRC4, NLRP3 (**D**, *Nlrc4*$^{-/-}$*Nlrp3*$^{+/-}$ (circles) and *Nlrc4*$^{-/-}$*Nlrp3*$^{-/-}$ (open circles)) and Caspase-11 (**E**, *Nlrc4*$^{-/-}$*Casp11*$^{+/-}$ (circles) and *Nlrc4*$^{-/-}$*Casp11*$^{-/-}$ (open circles)) are not involved in restriction of mLN colonization. Depicted are counts of all *S*. Tm (dark blue, selected for with Streptomycin) and specifically of the WITS (light blue, selected for with Kanamycin, 5% of the inoculum) (A-E, left panels). A-E, right panels: Number of WITS tags detected in mLN of mice which are shown in the left panels. Only mice with detectable WITS in the mLN (plating) were included in the analysis, remaining samples were set to 0. Each circle represents one mouse. Combined data of at least three independent experiments (see respective main figures). Dotted line: detection limit. Grey line: Median. Statistical analysis: Mann-Whitney-U Test, p-values indicated, ns: $p \geq 0.05$.

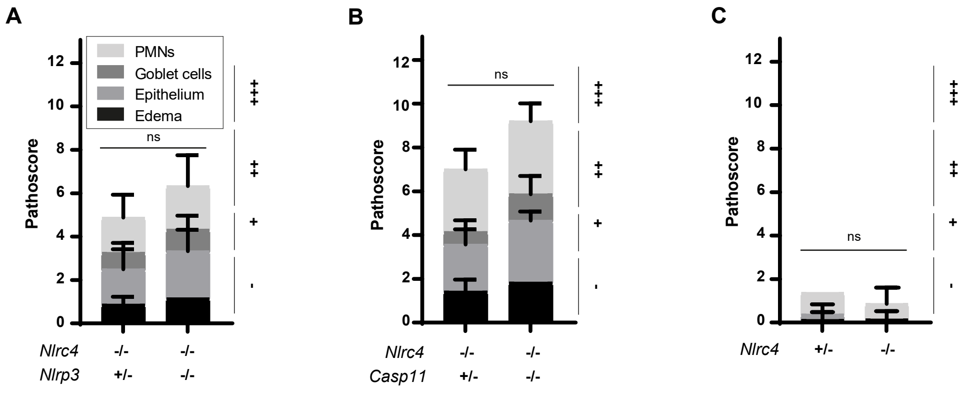
A
B
C
PMNs
Goblet cells
Epithelium
Edema
Pathoscore
ns
+++
++
+
-
Nlrc4 -/- -/-
Nlrp3 +/- -/-
Nlrc4 -/- -/-
Casp11 +/- -/-
Nlrc4 +/- -/-

Figure S4

Figure S4: Histopathological analysis. Histopathological scores of cecae of **A** *Nlrc4*$^{-/-}$*Nlrp3*$^{+/-}$ (n=8) and *Nlrc4*$^{-/-}$*Nlrp3*$^{-/-}$ (n=6) mice shown in Fig S3D, **B** *Nlrc4*$^{-/-}$*Casp11*$^{+/-}$ (n=7) and *Nlrc4*$^{-/-}$*Casp11*$^{-/-}$ (n=6) mice shown in Fig S3E and **C** of *Nlrc4*$^{+/-}$ (n=8) and *Nlrc4*$^{-/-}$ (n=7) mice infected with *S*. Tm$^{\Delta 4}$ shown in Fig 3B. H&E stained sections were scored for submucosal edema, epithelial integrity, goblet cell loss and PMN infiltration. Symbols on the right indicate inflammatory states from "-", no inflammation, to "+++", strongly inflamed. Statistical analysis: Two-way ANOVA with Tukey`s correction, ns: $p \geq 0.05$.

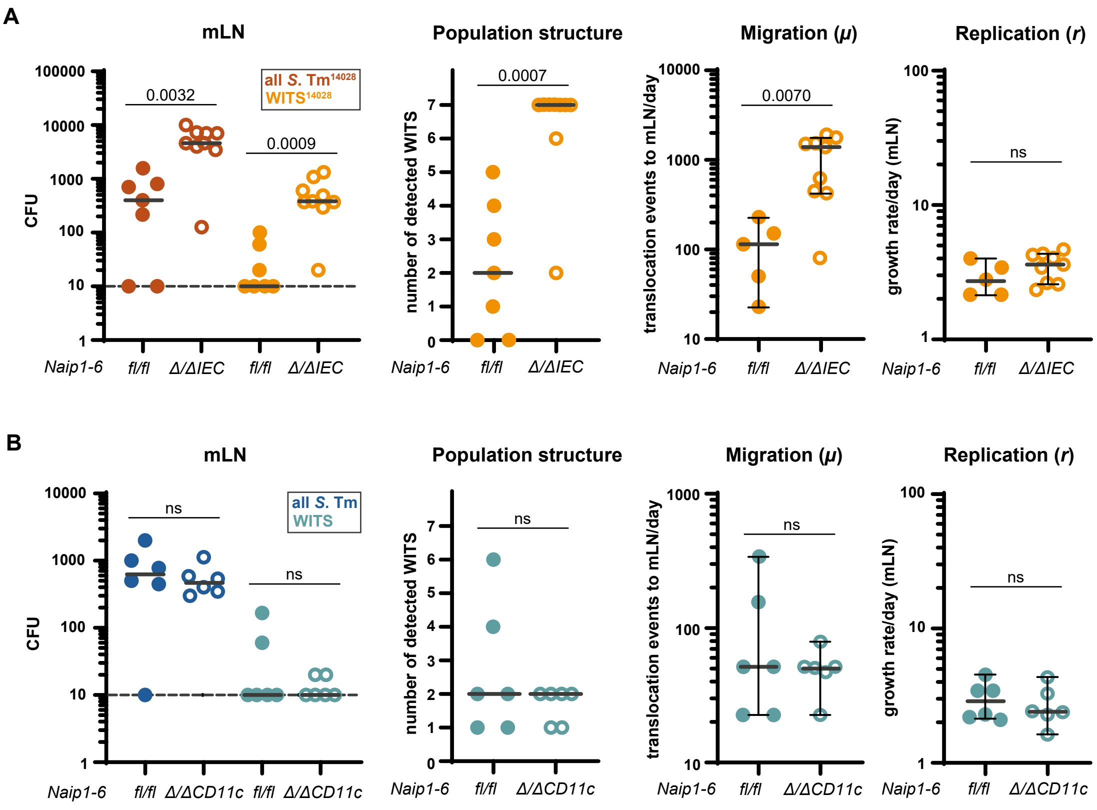
A
mLN
Population structure
Migration (μ)
Replication (r)
all S. Tm14028
WITS14028
CFU
0.0032
0.0009
0.0007
0.0070
ns
number of detected WITS
translocation events to mLN/day
growth rate/day (mLN)
Naip1-6
fl/fl
Δ/ΔIEC
B
all S. Tm
WITS
Δ/ΔCD11c

Figure S5

Figure S5: DC NAIP/NLRC4 is dispensable for restriction of *S*. Tm mLN loads. A Kanamycin pretreated mice were orally infected with $5x10^7$ CFU *S*. Tm^{14028}. Streptomycin pretreated mice were orally infected with $5x10^7$ CFU *S*. Tm. *S*. Tm counts in the mLN at 24 hpi, number of WITS tags in the mLN, *S*. Tm migration rate μ to the mLN were increased in *Naip1-6*$^{\Delta/\Delta IEC}$ mice (open circles) compared to *Naip1-6*$^{fl/fl}$ littermates (circles), whereas the replication rate *r* was not affected. Depicted are counts of all *S*. Tm (brown, selected for with Kanamycin) and specifically of the WITS (orange, selected for with Chloramphenicol, 5% of the inoculum). **B** *S*. Tm counts in the mLN at 24 hpi, number of WITS tags in the mLN, *S*. Tm migration rate μ to the mLN, and replication rate *r* within the mLN were not altered in *Naip1-6*$^{\Delta/\Delta CD11c}$ mice (open circles) compared to *Naip1-6*$^{fl/fl}$ littermates (circles). Depicted are counts of all *S*. Tm (dark blue, selected for with Streptomycin) and specifically of the WITS (light blue, selected for with Kanamycin, 5% of the inoculum). Each circle represents one mouse. Combined data of two (A) or five (B) independent experiments. Dotted line: detection limit. Grey line: Median, for μ and *r*, 95%-Confidence Intervals are indicated. Statistical analysis: Mann-Whitney-U Test, p-values indicated, ns: $p \geq 0.05$.

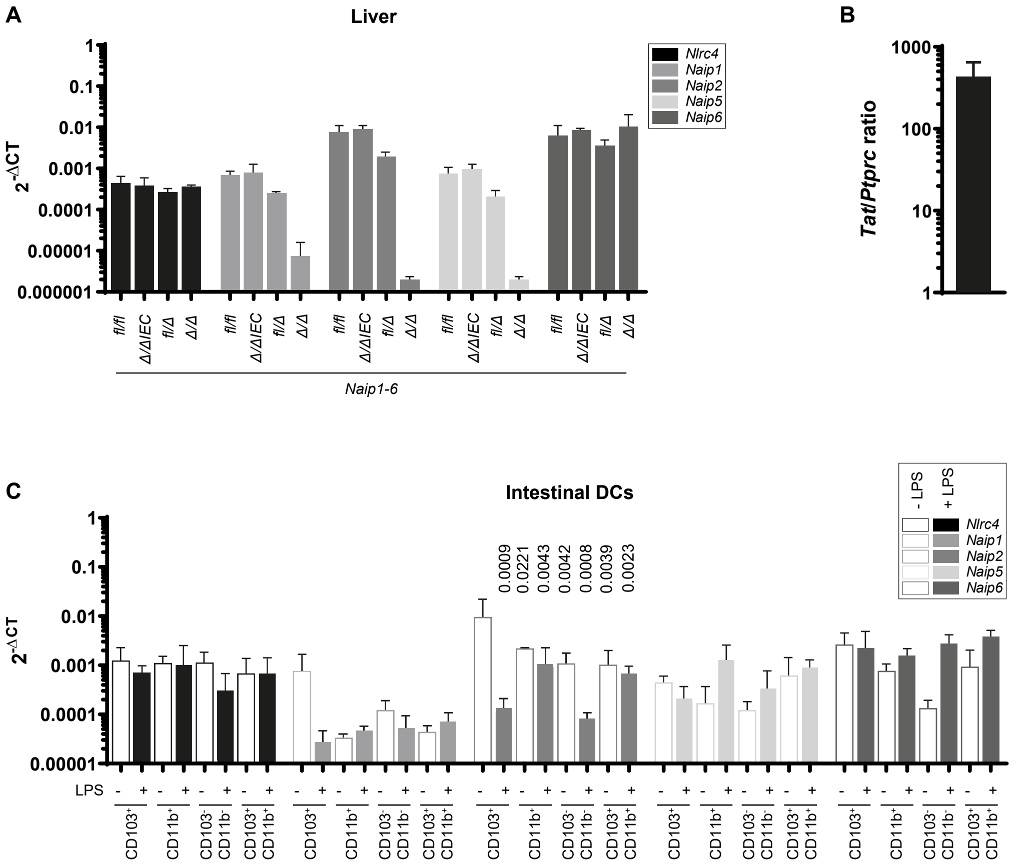
A
Liver
B
C
Intestinal DCs
Nlrc4
Naip1
Naip2
Naip5
Naip6
2-ΔCT
fl/fl
Δ/ΔIEC
fl/Δ
Δ/Δ
Naip1-6
Tat/Ptprc ratio
- LPS
+ LPS
LPS
CD103+
CD11b+
CD103- CD11b-
CD103+ CD11b+
0.0009
0.0221
0.0043
0.0042
0.0008
0.0039
0.0023

Figure S6

Figure S6: NAIP/NLRC4 inflammasome components are expressed at low levels in non-barrier, non-lymphoid tissues, and in intestinal DCs. A *Naip*/*Nlrc4* transcripts can be detected at low levels in liver tissue. Quantification of transcript expression for *Nlrc4*, *Naip1*, *Naip2*, *Naip5* and *Naip6* in the liver of *Naip1-6*$^{fl/fl}$, *Naip1-6*$^{\Delta/\Delta IEC}$, *Naip1-6*$^{fl/\Delta}$ and *Naip1-6*$^{\Delta/\Delta}$ mice depicted as $2^{-\Delta CT}$ values. Expression levels were normalized to *Actb*. **B** For the samples depicted in A, expression levels of *Tat* as hepatocyte marker, and *Ptprc*, as a marker for immune cells, were compared to estimate the relative contribution of both cell types to the transcripts within the respective tissue. **C** Intestinal DCs express low levels of *Naip*/*Nlrc4*. This expression is not increased upon exposure to the proinflammatory stimulus LPS. Quantification of transcript expression for *Nlrc4*, *Naip1*, *Naip2*, *Naip5* and *Naip6* in sorted DCs from the cecum of PBS ("-")- or 5 µg LPS ("+")-injected wildtype mice (1h after injection) depicted as $2^{-\Delta CT}$ values. Expression levels were normalized to *Actb*. n=3. Mean and standard deviation plotted. Statistical analysis: two-way ANOVA with Tukey`s correction. P-values indicated in B in comparison to PBS-treated CD103^{+} DCs.

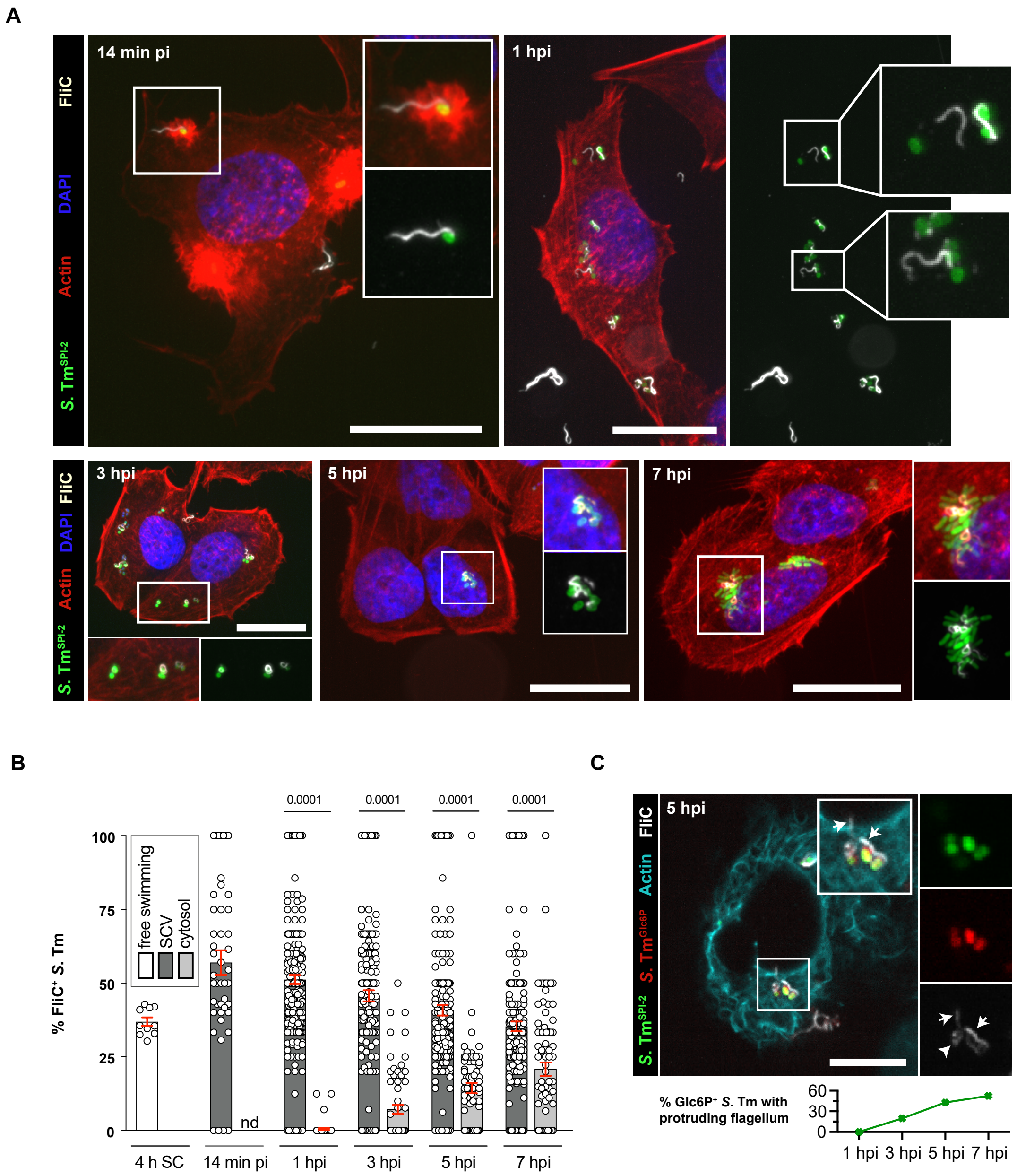
A
14 min pi
1 hpi
3 hpi
5 hpi
7 hpi
FliC
DAPI
Actin
S. Tm^SPI-2
B
% FliC+ S. Tm
free swimming
SCV
cytosol
0.0001
0.0001
0.0001
0.0001
100
75
50
25
0
nd
4 h SC
14 min pi
1 hpi
3 hpi
5 hpi
7 hpi
C
5 hpi
FliC
Actin
S. Tm^Glc6P
S. Tm^SPI-2
% Glc6P+ S. Tm with protruding flagellum
60
30
0
1 hpi
3 hpi
5 hpi
7 hpi

Figure S7

Figure S7: *S*. Tm carries flagella during invasion, within the SCV, and inside the host cell cytosol. A Flagellated *S*. Tm can be detected during early HeLa cell invasion (14 mpi, $n^{total\ \textit{S.}\ Tm}$=370) and persist until later infection times (1 hpi, $n^{total\ \textit{S.}\ Tm}$=2443; 3 hpi, $n^{total\ \textit{S.}\ Tm}$=1328); 5 hpi, $n^{total\ \textit{S.}\ Tm}$=1403; 7 hpi, $n^{total\ \textit{S.}\ Tm}$=1596). **B** Over the time course of HeLa cell infection, a subpopulation of flagellated *S*. Tm escaped from the SCV compartment (dark grey) to the cytosol (light grey). The fraction of flagellated *S*. Tm in the inoculum (“4 h SC”, white) provides a baseline of flagella expression. Data is depicted as mean and SEM. Quantifications are based on immunofluorescence staining for FliC and the use of different fluorescent reporter constructs (SCV, p*ssaG*-GFPmut2 for SPI-2-GFP (*S*. $Tm^{SPI\text{-}2\text{-}GFP}$); cytosol, pCK100 for Glc6P-mCherry (*S*. $Tm^{localizer}$)). **C** *S*. Tm carrying protruding flagella are found in the cytosol more frequently as the infection progresses ($n^{total\ Gl6P+\ flagellated\ \textit{S.}\ Tm}$=638). White arrows, elongated flagella; white arrowheads, flagella coiled around *S*. Tm. Scale bars, 20 µm.

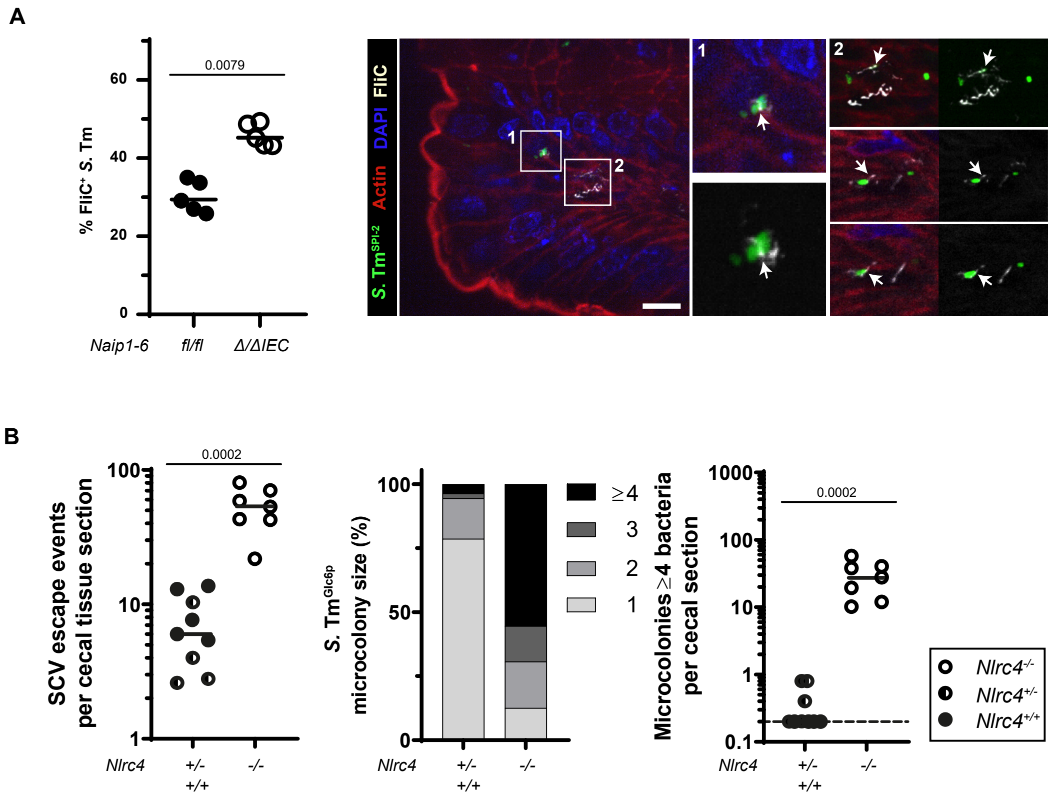
A
0.0079
% FliC+ S. Tm
Naip1-6
fl/fl
Δ/ΔIEC
S. Tm SPI-2 Actin DAPI FliC
1
2
B
0.0002
SCV escape events per cecal tissue section
Nlrc4
+/- +/+
-/-
S. Tm Glc6p microcolony size (%)
≥4
3
2
1
0.0002
Microcolonies ≥4 bacteria per cecal section
Nlrc4-/-
Nlrc4+/-
Nlrc4+/+

Figure S8

Figure S8: The relative abundance of flagellated *S*. Tm within IECs is restricted by NAIP/NLRC4. A IEC-specific deletion of NAIP1-6 (*Naip1-6*$^{\Delta/\Delta IEC}$; open circles; $n^{total\ S.\ Tm}$=776) leads to an increase of flagellated *S*. Tm at 12 hpi within the cecal mucosa (*Naip1-6*$^{fl/fl}$; circles; $n^{total\ S.\ Tm}$=238). Quantifications are based on immunofluorescence staining for FliC. **B** This correlates with an increase in cytosolic *S*. Tm at 18 hpi within IECs of *Nlrc4*$^{-/-}$ mice (open circles) compared to *Nlrc4*$^{+/-}$ littermates (half-closed circles) and wildtype mice (*Nlrc4*$^{+/+}$, circles) as quantified by counting of *S*. Tm$^{Glc6p\text{-}mCherry}$ in fluorescently labelled cecal tissue sections. Black bar, median. Statistical analysis, Mann-Whitney-U Test, p-value indicated. Scale bar, 10 µm.

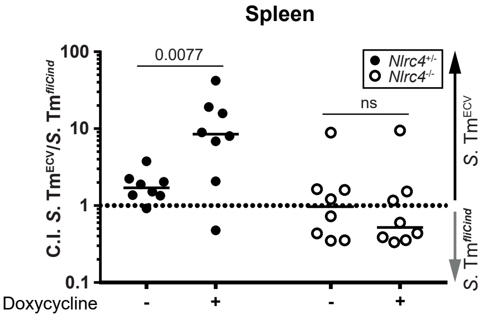

Spleen
C.I. S. Tm^ECV/S. Tm^fliCind
100
10
1
0.1
0.0077
ns
Nlrc4+/-
Nlrc4-/-
S. Tm^ECV
S. Tm^fliCind
Doxycycline
-
+
-
+

Figure S9

Figure S9: When FliC expression in phagocytes is forced, NAIP/NLRC4 restricts *S*. Tm within the spleen. *Nlrc4*$^{+/-}$ (circles) and *Nlrc4*$^{-/-}$ (open circles) mice were iv infected with 10^4 CFU of a 1:1 mix of *S*. Tm^{ECV} and *S*. Tm^{fliCind}. At 17 hpi, 100 µl PBS (“-”, control) or 0.8 mg Doxycycline in 100 µl PBS (“+”) were iv injected to induce FliC expression. Induction of FliC expression leads to NLRC4-dependent elimination of *S*. Tm. The competitive index (C.I.) was normalized to the relative abundance of the two strains in the inoculum. The dotted line indicates a C.I. of 1, where none of the strains has an advantage. A C.I. > 1, indicates an advantage for *S*. Tm^{ECV}, a C.I. < 1 an advantage for *S*. Tm^{fliCind}. Each circle represents one mouse. Combined data of three independent experiments. Statistical analysis: Two-way ANOVA with Tukey`s correction, p-values indicated, ns: $p \geq 0.05$.
